# Exploring recent progress of molecular farming for therapeutic and recombinant molecules in plant systems

**DOI:** 10.1016/j.heliyon.2024.e37634

**Published:** 2024-09-07

**Authors:** Jothi Kanmani Bharathi, Preethika Suresh, Muthu Arjuna Samy Prakash, Sowbiya Muneer

**Affiliations:** a Department of Genetics and Plant Breeding, Faculty of Agriculture, Annamalai University, Annamalai Nagar, 608002, Tamil Nadu, India; b School of Bioscience and Biotechnology, Vellore Institute of Technology, Vellore, Tamil-Nadu, India; c Department of Horticulture and Food Science, School of Agricultural Innovations and Advanced Learning, Vellore Institute of Technology, Vellore, Tamil-Nadu, India

**Keywords:** Plant molecular farming, Biopharming, Plantibody, Plant-based vaccines, recombinant proteins

## Abstract

An excellent technique for producing pharmaceuticals called "molecular farming" enables the industrial mass production of useful recombinant proteins in genetically modified organisms. Protein-based pharmaceuticals are rising in significance because of a variety of factors, including their bioreactivity, precision, safety, and efficacy rate. Heterologous expression methods for the manufacturing of pharmaceutical products have been previously employed using yeast, bacteria, and animal cells. However, the high cost of mammalian cell system, and production, the chance for product complexity, and contamination, and the hurdles of scaling up to commercial production are the limitations of these traditional expression methods. Plants have been raised as a hopeful replacement system for the expression of biopharmaceutical products due to their potential benefits, which include low production costs, simplicity in scaling up to commercial manufacturing levels, and a lower threat of mammalian toxin contaminations and virus infections. Since plants are widely utilized as a source of therapeutic chemicals, molecular farming offers a unique way to produce molecular medicines such as recombinant antibodies, enzymes, growth factors, plasma proteins, and vaccines whose molecular basis for use in therapy is well established. Biopharming provides more economical and extensive pharmaceutical drug supplies, including vaccines for contagious diseases and pharmaceutical proteins for the treatment of conditions like heart disease and cancer. To assess its technical viability and the efficacy resulting from the adoption of molecular farming products, the following review explores the various methods and methodologies that are currently employed to create commercially valuable molecules in plant systems.

## Highlights

• Plant molecular farming offers economic advantages for producing a variety of products, including pharmaceuticals and industrial proteins, with fewer restrictions.
• Plants can be engineered to produce antibodies for targeted treatment, showing promise in fighting infections.
• Regulatory complexities pose a barrier to broad adoption of plant systems for therapeutic protein production, while non-pharmaceutical proteins benefit from easier commercialization.
• Research is needed to identify mid-to-high-value by-products from biomass leftover in the manufacturing process, enhancing process income and sustainability.

## Graphical abstract

**Figure:**
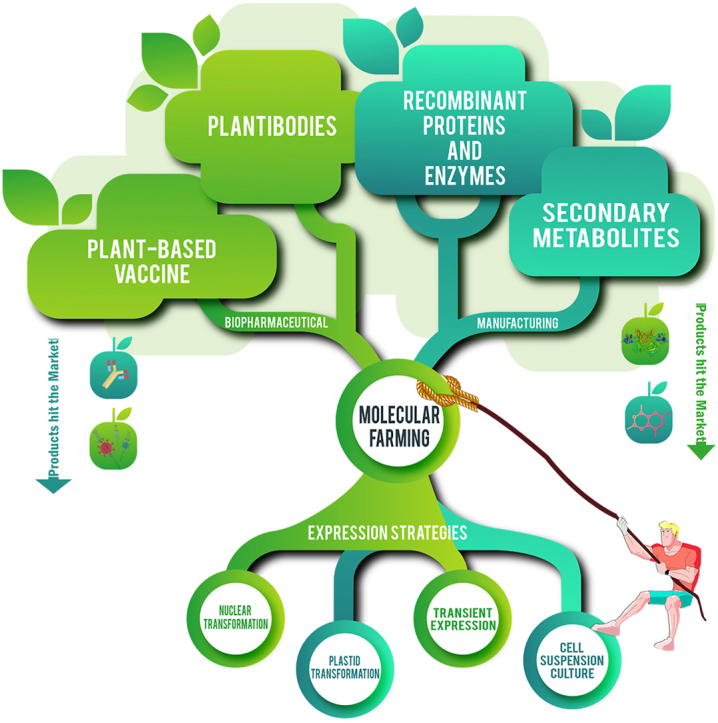

**Table:** Abbreviations

BTV	Bluetongue virus
BVDV	Bovine viral diarrhea virus
CCHFV	Crimean-Congo haemorrhagic fever virus
CHIKV	chikungunya virus
CoOMT	Columbamine O-methyltransferase
CP	Capsid protein
CPMV-HT	Cowpea mosaic virus based HyperTrans
CTB	Cholera toxin
CTB-GFP	Cholera toxin B-subunit and green fluorescent protein
EIC	Ebola immune complex
EIII	E glycoprotein domain III
ETEC	Enterotoxigenic *E. coli*
EHEC	Enterohemorrhagic *E. coli*
FaeG	F4 fimbrial adhesin
Fc	Fragment crystallizable
FhCMB	Fraunhofer USA Center for Molecular Biotechnology
FimA	Fimbrial protein fimbrillin
Flu	Influenza
FMDV	Foot and mouth disease virus
GCase	Glucocerebrosidase
GD	Gaucher disease
GP	Glycoprotein
GTFs	Glycosyltransferases
H1N1	Human influenza strains
H2GP1	Heavy chain-GP1 fusion protein
H5N1	Avian influenza virus
HAs	Haemagglutinin
HBsAg	Hepatitis B surface antigen
HBV	Hepatitis B virus
H-chain	Heavy chain
hGH	Human growth hormone
HIV	Human immunodeficiency virus
HPV	Human papillomavirus
HR	Hairy roots
hSA	Human serum albumin
IBDV	Infectious bursal disease virus
IBV	Infectious bronchitis virus
LFM	Leaf fresh mass
LT-B	Heat-labile enterotoxin B subunit
mAb	Monoclonal antibody
MF	Molecular farming
MOMP	Major outer membrane protein
NaV	Narita 104 virus
NDV	Newcastle disease virus
NV	Norwalk virus
PA	Protective antigen
PMF	Plant molecular farming
PMPs	Plant-made pharmaceuticals
PRRSV	Porcine reproductive and respiratory syndrome virus
PzE	Plant-produced zE
QVLP	Quadrivalent virus-like particle
r-antibody	Recombinant antibody
r-antigen	Recombinant antigen
RBD	Receptor binding domain
RGP-RTB	Rabies Glycoprotein, Ricin Toxin B Chain
RHDV	Rabbit hemorrhagic disease virus
rNV	Recombinant Norwalk virus
r-protein	Recombinant protein
RV	Rotaviruses
SARS-CoV-2	Severe acute respiratory syndrome coronavirus 2
sIgA	Secretory immunoglobulins A
sIgM	Secretory immunoglobulins M
TGEV	Transmissible gastroenteritis virus
VLP	Virus-like particles
WNV	West Nile virus
WT	Wild type
zE	ZIKV envelope E
ZIKV	Zika virus

## 1 Introduction

Molecular farming (MF) uses plants as factories to produce valuable proteins like pharmaceuticals through genetic modification [1]. The first recombinant human proteins, growth hormone, and insulin were created in the 1980s by the bacteria *Escherichia coli*, which marked the beginning of molecular farming. The first industrial MF process was introduced in 1996 and featured the synthesis of protein-based technological chemicals in maize seeds [2]. Plants have always given humans useful products, but over the past 30 years, they have also been used to create small molecules and recombinant proteins (r-proteins). Due to the expansion of the global bioeconomy, this once-niche field has developed and is now the residence of renewable energy sources, chemical building blocks, and polymers. All these uses fall under the category of "plant molecular farming" (PMF). The market for PMF products was estimated to be worth $116.1 million in 2021 and is predicted to grow to $182.9 million by 2031 [3]. The use of pharmacologically active proteins in a range of clinical therapies has undergone a revolution because of research in recent years. It is crucial to choose which system gives the greatest benefits for the synthesis of the r-protein because the majority of genes may be expressed in a variety of systems [4]. PMF hasn't been widely adopted by industry despite its promise to improve the viability of biologics manufacturing [5]. Human biopharmaceuticals, dietary supplements, recombinant antibodies, biodegradable polymers, recombinant vaccine subunits, and several other nonpharmaceuticals have all been successfully fabricated in plants in recent decades. Several therapeutic products, such as diagnostic proteins (enzymes and antibodies), protein replacements (insulin for diabetics, factor VIII for hemophiliacs), immune system stimulators/suppressants (colony-stimulating factors, interferons, and interleukins), adhere proteins and biopolymers for surgical uses, and growth factors (GF) can all be produced in plants [6]. Over a hundred distinct heterologous proteins, including hormones, antigens, enzymes, anticoagulant peptides, antibodies, and molecular transporters been produced in various plant systems since 1986, whereas human growth hormone (hGH), the first significant recombinant pharmaceutics, was initially developed in transgenic tobacco and sunflower cells [7]. However, the earliest commercially useful recombinant egg protein avidin was not produced in transgenic maize until 1997 [8]. Whereas entire crops like cereals and tobacco are frequently used in MF, additional plant-based systems are also included in this technique such as algae, moss, aquatic plants, and even *in vitro* gene expression networks made from plant cells [1]. It has been largely facilitated by their ability to make post-translational changes that ensure proper folding and keeping of structural and functional integrity of these r-proteins [9]. PMF technologies are getting attention from researchers throughout the world because of the continuous rise in the urges for r-proteins in terms of quantity, quality, and variety [10,10]. Human serum albumin (hSA), insulin, and HIV-neutralizing antibodies are instances of plant-made pharmaceuticals (PMPs) with international markets. Because of the widespread prevalence of diabetes, including a major undersupplied market in Asia, human insulin is in great demand. This insulin shortage might be filled by plant production at a cost that diabetics in this area could afford [11]. A rice-derived hSA called Optibumin was produced by InVitria, a branch of Ventria Bioscience, and it has already achieved mainstream success [12]. In 2012, the market saw the release of the first plant-based recombinant medication licensed for use by people [2]. Plants are now a desirable production platform that can even attain economically pertinent production in a relatively short time because of improvements in PMF techniques over the last decades [10]. This review on MF in plant systems stands out for its focus on recent advancements, comprehensive coverage of plant-based vaccine and therapeutic protein production, emphasis on safety and regulatory aspects, discussion of economic viability, sustainability, and future perspectives.

## 2 Tools and approaches of MF

### 2.1 Expression strategies for MF

**Fig. 1:**
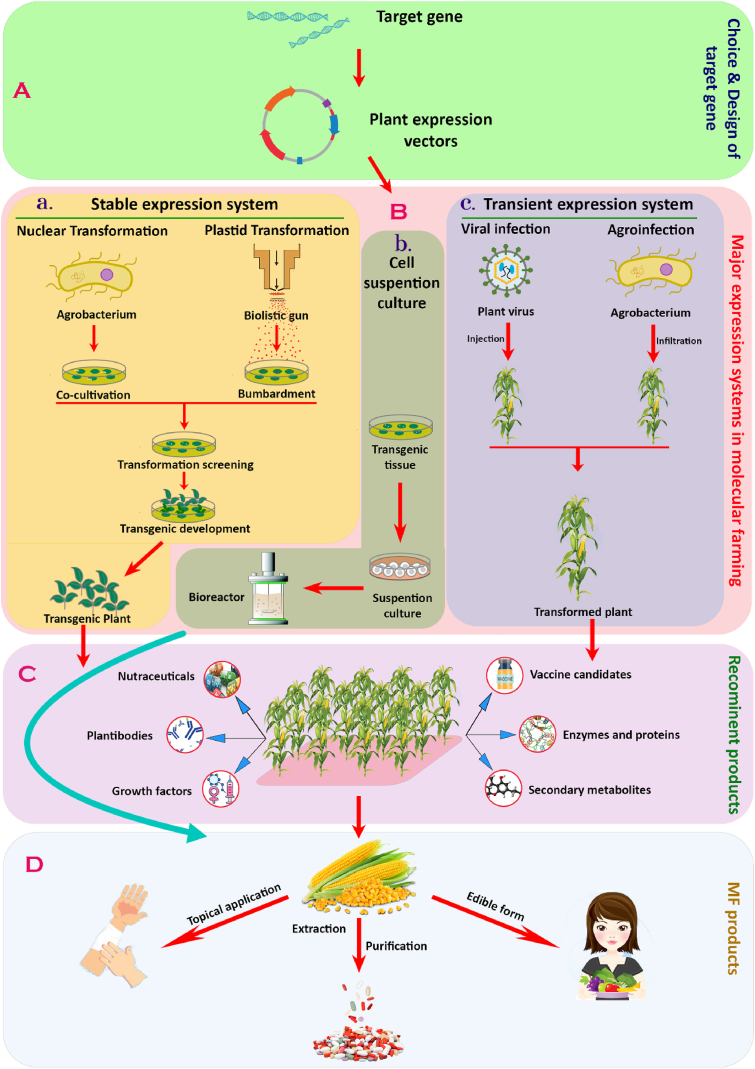
Schematic delineation of plant molecular farming. A. Selection of candidate gene and expression vector design. B. Available plant-based production systems: a. Stable expression system produced by either nuclear transformation using agrobacterium, or chloroplast transformation using gene gun. b. Cell suspension of transgenic callus was cultured in a bioreactor. c. Transient/Temporary expression system produced by plant viral injection and agrobacterium infiltration. C. Popular recombinant products produced in the PMF system. D. Form of MF products utilized for commercialization.

Chromosomal incorporation of a heterologous gene is necessary for plant transformation, and it is getting easier. Technical and practical challenges must be resolved, such as creating potential transformation methods for all important crop species [13]. Recently, there are three methods for creating PMF products (Fig. 1).

#### 2.1.1 Stable/permanent expression

This approach needs a technique for introducing the foreign genes into the plant cells that will allow them to be taken up and steadily integrated into the host nuclear genome, by utilizing *Agrobacterium tumefaciens* (for dicots) or particle bombardment (for monocots) [13]. Long-term recombinant product synthesis is possible because these transformations produce stable recombinant products that can be passed down from generation to generation [14]. Other classifications of stable/permanent expression systems include the following.

a. *Nuclear transformation*: All the items that are now on the market were developed mostly by using the nuclear transformation of plant species. This approach has several benefits. The protein product often accumulates inside the seeds when applied to a crop species like grains. They are then collected in dry condition and kept in storage until processing is finished. Also, this method may be used for huge areas at the least expensive. Even so, developing a permanent transgenic plant might take months or even years based on the plant type employed for r-protein production. The ability of some crops, like maize, which hybridize with local species or food crops is another drawback of this approach [14].
b. *Plastid transformation*: This process relies on the homologous recombination of target genes into chloroplast genome sequences. The nuclear transformation has many drawbacks, including the potential for transgene escape by amphimixis. Plastid transformation extends multiple benefits over nuclear transformation. Several species pass their plastids through their maternal tissue, therefore the absence of chloroplasts in most pollen species minimizes environmental issues. The drawback of this approach is that product stability would deteriorate with time in any fresh tissue MF system [15].

#### 2.1.2 Transient expression

Transgenic plants are usually utilized to produce recombinant products. However, it requires a huge time to develop transgenics, and protein expression is relatively less instead, r-proteins can be expressed quickly at elevated levels using transient expression methods. This is because agroinfiltration employing biolistics, viral vectors, or agrobacteria can be done. Agroinfiltration makes use of vacuum infiltration to introduce Agrobacteria into healthy leaf tissue. Incorporating required genes into the host cells simplifies protein production in leaf tissues. The target gene is usually regulated by a strong sub-genomic promoter and cloned into the genome of a plant pathogenic virus. The r-protein is then produced by plants that have been infected with pathogenic recombinant viral transcripts. The foreign genetic material does not incorporate into the plant's genome during temporary expression. Transient expression can be performed in experimental trials prior to the prolonged and expensive stable transformation [16].

#### 2.1.3 Cell suspension culture

Transgenic calluses were suspended with liquid growth media in bioreactors. This method enables the large-scale production of MF products [17]. Each strategy offers unique benefits and limitations in terms of protein expression, scalability, production efficiency, and regulatory considerations. Table 1 highlights the advantages, drawbacks, and strategies for overcoming challenges associated with nuclear transformation, plastid transformation, transient expression, and cell suspension systems.

**Table 1:** Comparison of advantages and limitations of expression strategies in plant systems.

Expression strategies	Advantages	Drawbacks	Strategies for overcome the problems
Nuclear transformation	• Heritable expression of recombinant proteins. • Ability to produce proteins in seeds for easy storage and processing. • Cost-effective production on a large scale. • Potential for proximity to target markets due to widespread cultivation of crop species like rice and corn. • Established method for producing all products available in the market. • Suitable for long-term recombinant protein production [14].	• Time-consuming development of stably transformed plants. • Potential for crossbreeding with native species or food crops. • Higher manual labour requirements and lower yields in some cases. • Concerns about transgene escape and outcrossing. • Limited scalability for producing large quantities of products. • Challenges in regulating protein stability over time and post-harvest processing [14].	• **Enhance Transformation Efficiency**: Improving the efficiency of gene transfer methods is crucial. This can be achieved by optimizing transformation protocols, selecting appropriate vectors, and enhancing the integration of foreign genes into the plant genome. Increasing the success rate of stable transformation will lead to higher yields of recombinant proteins. • **Choose Host Plants Wisely**: Selecting plant species that are well-suited for stable transformation and have high protein expression capabilities is essential. Opting for crop species with established transformation protocols and favourable characteristics for protein production can streamline the process and improve overall efficiency. • **Design High-Expression Constructs**: Developing expression constructs that promote high levels of protein expression and stability in plant cells is key. Utilizing strong promoters, suitable signal peptides, and codon optimization can enhance protein production efficiency and ensure robust expression of the desired recombinant proteins. • **Streamline Downstream Processing**: Optimizing downstream processing steps, such as protein extraction, purification, and formulation, is critical. Implementing efficient purification techniques tailored to plant-derived proteins can improve product quality, increase yield, and reduce production costs. • **Control Transgene Expression**: Implementing strategies to regulate transgene expression levels can prevent unintended effects on plant physiology. Using inducible promoters or tissue-specific expression systems can help control protein production, minimize potential negative impacts on plant growth, and ensure proper protein expression. • **Manage Environmental Concerns**: Addressing environmental concerns related to transgenic plants is essential. Implementing containment measures, such as mechanical detasseling or genetic male sterility, can prevent gene flow and mitigate the risk of transgene escape, ensuring environmental safety and regulatory compliance [56].
Plastid transformation	• Prevention of transgene escape through amphimixis. • Inheritance through maternal tissue in most species. • Reduced environmental concerns due to the absence of chloroplasts in most pollen. • Potential for high levels of protein expression, with reports of up to 70 % of total soluble proteins. • Ability to produce pharmaceutical proteins in high concentrations. • Superior solution compared to nuclear transformation in certain aspects [196].	• Changes in protein stability over time in fresh tissue systems, requiring precise timing for extraction and purification. • Limited scalability for producing large quantities of products. • Challenges in post-harvest processing due to the need for extraction and purification at specific times. • Restrictions on the use of certain crops like tobacco, which is a highly regulated crop and not edible. • Concerns about the system's capability to produce edible vaccines or large quantities of products. • Potential limitations in terms of yield and effectiveness compared to other transformation methods [196].	• Implement strict timing protocols for extraction and purification to maintain protein stability over time. • Explore alternative crops or species that are more suitable for plastid transformation and have higher scalability for production. • Develop methods to enhance protein yield and effectiveness within the plastid transformation system. • Investigate ways to improve post-harvest processing techniques to optimize protein extraction and purification. • Conduct research on enhancing the capabilities of plastid transformation for producing edible vaccines and large quantities of products. • Collaborate with regulatory bodies to address concerns about using regulated crops like tobacco and explore options for utilizing other edible plant species for plastid transformation [61].
Transient expression	• Rapid production of recombinant proteins in a matter of days, allowing for quick response to emergencies. • Ability to close production gaps quickly in response to new antigen sequences. • Flexibility to produce various types of proteins, such as subunit vaccines, in raw or partially processed fruits and vegetables. • Suitable for developing emergency vaccines and diagnostics, such as those for novel influenza virus strains and SARS-CoV-2. • Speedy production capacities that are crucial during emergency scenarios when other drug production cannot be delayed. • Effective strategy for producing diagnostics and therapeutics against emerging diseases like SARS-CoV-2 [14].	• Limited scalability for large-scale production compared to stable transformation methods. • Challenges in producing proteins that need to be consumed in large quantities. • Requirement for immediate processing of the product as storage can lead to plant tissue degradation. • Potential issues with protein stability and expression levels compared to stable transformation methods. • Not suitable for long-term production of certain proteins that require sustained expression.	• **Optimization of Expression Conditions**: Fine-tuning the parameters of transient expression, such as the choice of promoter, vector design, infiltration method, and post-translational modification signals, can help improve protein yield, stability, and consistency. • **Enhancing Protein Stability**: Incorporating protein stabilization motifs or chaperones in the expression construct can improve the stability and correct folding of transiently produced proteins, reducing the risk of degradation or misfolding. • **Batch-to-Batch Consistency**: Implementing quality control measures and standardized protocols for transient expression can help ensure consistency in protein expression levels and quality across different plant batches or experiments. • **Scale-Up Strategies**: Developing efficient scale-up strategies for transient expression, such as optimizing production facilities, automation of processes, and implementing robust purification methods, can facilitate large-scale manufacturing while maintaining protein quality and yield. • **Regulatory Compliance**: Engaging with regulatory authorities early in the development process, conducting thorough risk assessments, and ensuring compliance with relevant regulations can help address regulatory concerns associated with products derived from transient expression systems. • **Cost-Effectiveness**: Conducting cost-benefit analyses, optimizing production processes for efficiency, and exploring novel technologies or platforms that enhance the cost-effectiveness of transient expression can help mitigate the economic challenges associated with this expression system. • **Hybrid Approaches**: Combining transient expression with stable transformation methods, such as using transient systems for rapid protein screening and optimization before transitioning to stable transgenic production for large-scale manufacturing, can leverage the strengths of both systems [43].
Cell suspension	• Ability to produce high-value bioactive compounds in some plant species. • Ease of scale-up for manufacturing purposes. • Aseptic conditions can be maintained during growth using traditional fermentation technology. • Simplified purification process due to no tissue disruption requirement and low contaminating proteins. • Regulatory requirements similar to well-characterized production systems based on microbial and mammalian cells. • Reduction in heterogeneity of proteins and sugar in terms of cell type and size uniformity. • Faster system compared to transgenic plant production, with cell lines being produced in a few months. • Potential to become the preferred choice among plant-based systems for producing high-value recombinant proteins due to combining benefits of other systems [17].	• Proteins produced in cell suspension cultures may not always fold correctly or undergo proper post-translational modifications, leading to reduced functionality or efficacy of the recombinant proteins. • Obtaining regulatory approval for products derived from cell suspension cultures may be more complex due to concerns about cell culture contaminants, potential adventitious agent presence, and the use of bioreactors in production, necessitating thorough compliance with regulatory requirements. • Prolonged culture periods in cell suspension cultures may lead to genetic changes or drift in cell lines, which can impact protein expression levels, stability, or other characteristics. Regular monitoring and maintenance of cell lines are essential to mitigate this risk. • caling up cell suspension cultures from small laboratory volumes to industrial-scale bioreactors can be challenging, requiring careful optimization of culture conditions, nutrient supply, and oxygenation to maintain optimal growth and protein production. • he initial investment and operational expenses associated with setting up and maintaining cell suspension cultures in bioreactors can be significant, potentially making this production system more costly compared to other expression platforms. • Recombinant proteins expressed in plant cell suspension cultures may not always be efficiently secreted into the supernatant, leading to variability in the amount of protein recovered. Some of the protein may be retained within the cells, affecting overall productivity and product quality [17].	• **Optimization of Culture Conditions**: Fine-tuning the culture conditions, including nutrient composition, pH levels, temperature, and aeration, can help improve cell growth and protein production in suspension cultures. Optimization of these parameters can enhance productivity and protein yields. • **Selection of High-Producing Cell Lines**: Identifying and selecting cell lines with high protein production capabilities can significantly improve the efficiency of molecular farming using suspension cultures. Screening and optimizing cell lines for specific proteins of interest can enhance production levels 5. • **Genetic Engineering**: Implementing genetic engineering techniques to enhance protein expression in cell suspension cultures can be beneficial. Strategies such as gene stacking, promoter optimization, and gene editing can boost protein yields and improve the efficiency of molecular farming 5. • **Bioreactor Design and Scale-Up**: Utilizing advanced bioreactor systems and scaling up suspension culture production processes can help overcome limitations in scalability and production volume. Designing bioreactors for optimal cell growth and protein expression can increase overall productivity 5. • **Purification and Downstream Processing**: Developing efficient purification and downstream processing methods for extracting and purifying recombinant proteins from cell suspension cultures is essential. Streamlining these processes can improve protein quality, yield, and overall production efficiency 5. • **Risk Mitigation Strategies**: Implementing biocontainment measures and risk mitigation strategies to prevent contamination and maintain the integrity of cell suspension cultures is important. Proper containment protocols and sterile techniques can help minimize the risk of cross-contamination and ensure product quality 5. • **Regulatory Compliance**: Ensuring compliance with regulatory guidelines and quality standards for products derived from cell suspension cultures is crucial. Adhering to regulatory requirements and conducting thorough quality control assessments can facilitate the approval and commercialization of molecular farming products [43].

### 2.2 Improving PMF product quality and yield

The large-scale cultivation of some species was previously unsuitable or not suggested for traditional agriculture which raises several practical concerns. In the case of platforms based on plant-derived products, expression persistence, and product biochemical activity are crucial. It can be challenging to assess how well an expression construct performs across species since the inherent level of activity and specificity of a promoter might vary depending on the genetic background [18].

#### 2.2.1 Targeted gene insertion

Particle bombardment or *Agrobacterium tumefaciens*, the conventional approach of gene transformation, produces random transgene integration. Variable expression levels result from position effects and varying transgene copy numbers caused by the inability to regulate the insertion event. Position effects comprise transgenic insertion near native regulatory elements and in genomic sites with distinct chromatin construction. Moreover, long-term transgene expression stability can be lowered by epigenetic alterations like methylation at the insertion site, which eventually results in a decline in productivity [19]. Site-specific nucleases (SSNs) provide a solution to the problems associated with random transgene incorporation. SSNs allow for the regulated integration of transgenes by creating a double-strand break (DSB) at a desired region. Because site-specific DNA insertion is not the desired result of DSB repairs in plants, it is still more difficult than SSN-induced indel creation. Although SSN-approach has not yet been employed especially for PMF programs, it has been documented in a few research involving a variety of species, such as arabidopsis, maize, barley, potato, rice, soybean, and tobacco [[20], [21], [22], [23], [24], [25]]. Additionally, Numerous benefits are provided by chloroplast genetic engineering (CGE) for large-scale production. Initially, it permits r-proteins to be expressed at a high level in plant chloroplasts, leading to higher yields of the target product. Furthermore, transgene confinement through maternal inheritance is made possible by CGE, which maintains genetic stability and stops transgene flow to wild populations. Because chloroplasts have several copies of the transgene and provide higher protein yields per plant cell, they are also a cost-effective option for protein expression. Additionally, this method is especially beneficial for leaf-based expression, offering a productive platform for r-protein production in plant leaves. Finally, the scalable nature of CGE makes it easier to produce huge amounts of r-proteins to satisfy market demands [26]. For example.

#### 2.2.2 Expression vector constructs

The production of r-protein with a substantial expression yield is one of the goals of MF. r-protein expression constructs need to be adjusted at every step, from transcript stability to protein stability, to produce significant expression yields. It has been proposed recently that r-protein accumulation is not solely influenced by the guanine-cytosine composition of the target gene. Codon–anticodon dynamics is another significant factor that could have a significant impact on translation efficacy [27]. Most plant expression vectors are chimeric structures made from repurposed plant viruses, including potato virus X (PVX), cowpea mosaic virus (CPMV), alfalfa mosaic virus, and tobacco mosaic virus (TMV). The newest virus-based vectors are disassembled vectors in which the virus's non-essential genes have been substituted with the coding sequences of target genes or genes that promote high translation. The MagnICON system, the most renowned transient expression system, depends on constructs containing a PVX and a TMV backbone. A well-liked substitute is the pEAQ-HT expression system, which is derived from the CPMV and a variation that enhanced plant transient expression by logically creating synthetic 5′ and 3′ untranslated sections, ultimately leading to the creation of the pHREAC expression system [28]. Dicotyledonous plants have a strong constitutive promoter of the generated RNA transcripts, and the most often used promoter for these plants is CaMV 35S. The machinery of the cell will cap the emerging RNA transcripts at the 5′ end, which is necessary for translation in concert with the poly (A) tail. Duplicating the enhancer region can increase the CaMV 35S promoter's activity. But in monocotyledonous plants, this promoter exhibits reduced activity; hence, the maize ubiquitin-1 promoter is a better substitute [29].

#### 2.2.3 Codon optimization

A recent study found that in tobacco BY-2 cells, codon optimization increased the output of a recombinant stem cell factor protein by 25 to 30-fold [30]. However, reports about the outcomes of codon optimization are similarly inconsistent. For instance, there was a minimal increase in protein yield when the interferon-γ or human erythropoietin genes were optimized for plant expression [31]. Similar findings were made when optimizing codon usage for the development of the HPV-16 L1 capsid protein in *N. benthamiana*. It was discovered that native genes with human codon usage performed better than those that were adjusted to represent plant codon usage [32]. It has been demonstrated recently that codon optimization may occasionally have an impact on the mRNA structural patterns required for efficient protein production. A deeper comprehension of codon usage and tRNA pool sizes in the source and host species can also help to create codon-harmonized sequences for improved translational efficiency [5].

#### 2.2.4 Suppression of endogenous proteins

Depleting the pool of endogenous seed storage proteins can increase (2–10 fold) the yield of r-proteins by reducing competition for translation, folding, and assembly in the endoplasmic reticulum. E.g. The production of r-proteins, such as cedar pollen allergen, was enhanced by decreasing the 13-kDa prolamin pool in rice endosperm by proteome rebalancing [33]. RNAi or the application of expression hosts with traditionally produced mutations in storage protein genes have been the usual methods used thus far to decrease natural seed storage proteins in PMF applications [34,35]. Targeting storage protein genes in sorghum, camelina, and wheat has been done using CRISPR/Cas9 [36,37]. It is possible to generate seed crops deficient for endogenous storage proteins through genome editing as hosts for PMF production, as their phenotype is deemed stable and they lack RNAi constructions, which lowers the possibility of transgene cassette interference.

#### 2.2.5 Metabolic engineering

Tobacco and other non-food/feed plants lessen the chance of product contamination in the food/feed chain. Still, some plants are unsafe since they can yield harmful substances, like the tobacco plant's alkaloid nicotine. Biopharmaceutical products must undergo purification procedures to guarantee the removal of tiny molecules and protein-based contaminants, lowering their limit of detection [5]. Therefore, it makes sense to develop a PMF chassis free of such potentially harmful substances. This objective has been accomplished in tobacco by deleting both alleles of each of the six genes that code for berberine bridge enzyme-like (BBL) proteins, which carry out the last oxidation process in the formation of nicotine. This method involved the use of CRISPR/Cas9, which led to 99.6 % lower nicotine levels. However, if important enzymes are targeted during secondary metabolism modulation, this could have unintended side effects [38]. Metabolite levels can be spatiotemporally adjusted by targeting relevant transcription factors, rather than directly modifying enzyme expression through gene deletion or overexpression [39]. Compared to transgenic silencing, transient silencing of CysP6 was shown to be a more effective method, as evidenced by a rise in the accumulation of the target r-protein, interleukin (IL)-10, in plants. Attempts to inhibit protease degradation in suspension cultures of cell lines that produce the 2F5 antibody resulted in a reduction in protease degradation and an increase in antibody accumulation. Proteases can be successfully silenced via RNAi; alternatively, plant-derived protease inhibitors, like cystatins and cysteine protease inhibitors, can be co-expressed [40,41].

#### 2.2.6 Cellular level modification

The creation of structurally specified glycans remains a difficult problem despite their recognized significance for r-proteins. However, a better knowledge of glycosylation pathways, current developments in analytical techniques, deconstructed vectors, targeted genome editing, and developing approaches for subcellular localization employing chimeric glycosyltransferases (GTFs) are assisting in the logical design of unique glycan biosynthesis paths. Plants are widely employed for the manufacture of r-proteins because they are especially open to glycoengineering techniques [42]. One of the most important post-translational modifications (PTMs) is glycosylation, which is essential for the clinical efficacy of several pharmacological proteins, including vaccine antigens and antibodies [43]. A range of homogenous human-type glycoforms has been synthesized in planta using four main methods. First, the removal of plant-specific enzymes that produce non-human glycan structures. The second is the appearance of mammalian GTFs and supporting proteins. Thirdly, optimizing the subcellular localization of glycosylation processes. Finally, there is the utilization of multigene vectors that are transiently expressed [44]. With significant effort, human-type glycosylation pathways have been introduced into plants to replace plant-specific glycan systems, and these glycoengineered plants can now produce human-like glycoproteins. In recent decades, promising glycoengineering techniques have made it feasible to humanize plant N-glycosylation, either by de novo creation of heterologous GTFs that are comparable to those found in humans or by inactivating the host plant's Indigenous GTFs [45]. Another approach of non-immunogenic glycosylation, based on RNA interference, regulates the amounts of fucosyl glycans and xylosyl and stops the addition of undesired residual sugars to biotherapeutic proteins [9]. By eliminating plant-specific glycan processing events and engineering glycosylation pathways, researchers were able to achieve the production of viral glycoproteins with glycan structures that closely resembled those found on native viral proteins. This glycan engineering approach aimed to enhance the immunogenicity and efficacy of recombinant viral glycoprotein vaccines by ensuring that the glycosylation patterns mimicked those of the wild-type virus [46]. Glycoengineering successful knockout of two β(1,2)-xylosyltransferase and four α(1,3)-fucosyltransferase genes in Nicotiana tabacum cv. SR-1 using CRISPR/Cas9 technology. This genetic modification led to the generation of NtFX-KO lines with altered N-glycosylation patterns, specifically reducing the presence of core α(1,3)-fucose on N-glycans. The engineered plants exhibited improved glycan profiles with primarily GnGn N-glycans, which are more similar to human-like glycosylation patterns [47]. The production of complex protein-based pharmaceuticals in PMF is optimized by glycoengineering, ensuring that proper protein folding, functionality, and safety for human use are achieved. Human-like N-glycan structures are produced in plants through glycoengineering, allowing for the enhancement of protein functionality, the reduction of immunogenicity, and the stabilization of recombinant proteins and peptides to prevent proteolytic degradation and improve overall yield and quality of the final product [29].

#### 2.2.7 Cell culture system for production of recombinant product

Since their introduction in 1902, suspension culture methods have a rich history. They are frequently used in the field of plant biotechnology as an easy-to-use tool for producing r-proteins, secondary metabolites, and other medicinal components on a massive scale. Numerous instances of plant suspension culture have been documented; proteins obtained through this technique comprise the anti-tumor necrosis factor antibodies, human glucocerebrosidase, Newcastle disease virus's HN protein, and recombinant α-galactosidase-A. The choice of host plant, form of plant material (e.g., callus or hairy root), components of the media, type of bioreactor, and operating technique are all crucial elements to optimize when it comes to suspension culture [48,49]. The development of large-scale manufacturing processes depends on the proper functioning of bioreactors. Air-lift reactors, bench-top bioreactors, membrane reactors, bubble column reactors, single-use bubble column reactors, rotating drum reactors, stirred tank reactors, wave and undertow reactors, and wave reactors are among the several types of bioreactors. Their volumetric productivity varies overall between 4.5 and 7.7 mg/L and 100–247 mg/L [50]. Scaled-up automatic micropropagation of huge quantities of shoot biomass under standardized conditions is possible with temporary immersion bioreactors, which periodically submerge biomass in liquid media. Using this approach, the yield of *Borrelia burgdorferi* (OspA) outer surface protein A was 108 mg/L (7.6 % TSP) [51], and fragment C of tetanus toxin accumulated to around 95 mg/L (8 % TSP) [52]. Another effective method for creating recombinant proteins is an air-lift type bioreactor, which produces the B subunit of the heat-labile toxin produced by *E. coli*, which accumulated to 0.36 % TSP in *Siberian ginseng* somatic embryos [53]. The first plant-produced, commercially available therapeutic protein was created in carrot cells cultured in a flexible, disposable bioreactor made of polyethylene, which is the most remarkable accomplishment to date for a tank bioreactor system [54]. Because of their affordability, adaptability, and safety, disposable bioreactor systems like stirred tank reactors or wave-mixed bioreactors have gained popularity in the biomanufacturing sector as a substitute for stainless steel bioreactors, which are used for mammalian cell cultures.

## 3 Plant species for producing recombinant products

The expression of r-proteins has been achieved through the use of over 100 plant species, tobacco offers one of the best possibilities for the commercial production of r-protein because it has an extended record of success as a crop platform in MF [55]. The well-established technique for transgenic and expression, high biomass yield, presence of a vast processing network, defective RNA silencing, and prolific seed production are the main benefits of tobacco. There is minimal chance that tobacco material will infect the food or feed chains. Because tobacco is not a food or feed crop [56]. Lettuce and lucerne are among the green vegetation being studied for MF. Due to its high dry biomass production per area and ability to be harvested up to 9 times a year, lucerne is very beneficial. Recombinant antibodies have been produced from both lucerne and lettuce. Additionally, lettuce has been looked into as a potential host for recombinant edible vaccine production [57]. r-proteins are fragile when synthesized in an aqueous environment, which leads to decreased yields in leafy crops, which is one of their biggest drawbacks. To extract adequate quantities of the product, the leaves must be processed promptly after harvest or dried or frozen for transportation [56]. Contrary to greens, as a result of seeds having the ideal biochemical conditions to encourage reliable protein accumulation, seeds may be stored for a prolonged period, even at ambient temperatures. Additionally, cereal seeds don't contain the phenolic substances found in tobacco leaves, which increases the effectiveness of the subsequent processing. Several legumes (soybean and pea) and cereals (wheat, rice, and maize) have been demonstrated for seed-based production of r-proteins [55]. A significant benefit of protein expression in vegetable and fruit crops is the potential of the edible parts to be used for the development of nutraceuticals, topical antibodies, and recombinant vaccines. These products can be produced from edible parts, which can be eaten raw, uncooked, or with a bit of processing. At least 3 clinical studies conducted for vaccines were produced from potato tubers, and transgenic potatoes have been used for the production of human milk, antibodies fusion proteins, and glucanases [58]. In addition to having a higher palatability than potatoes, tomatoes also have additional benefits such as a high biomass output and the ability to be grown in greenhouses for better containment. Bananas provide appealing delivery vehicles for edible vaccines, due to their widespread cultivation and consumption by both adults and children [59].

Transgenic trees have also been explored as potential platforms for producing r-proteins. Trees such as poplar and eucalyptus have been genetically engineered to express valuable proteins for various applications [60]. These transgenic trees offer advantages such as high biomass production, long-term protein expression, and the potential for large-scale production of r-proteins [61]. Pulses, which include crops like chickpeas, lentils, and beans, have not been extensively studied for r-protein production compared to other plant species. However, pulses have the potential to serve as alternative platforms for MF due to their nutritional value, ease of cultivation, and genetic tractability. Further research is needed to explore the feasibility of using pulses as hosts for producing r-proteins [60]. Oilseed plants, such as safflower, rapeseed, and sunflower, have been utilized for producing r-proteins due to their high protein content and ease of purification. These oilseed plants offer advantages such as high protein yield, self-pollination, and simplicity in protein extraction [62]. Researchers have successfully expressed pharmaceutical proteins in oilseed plants, demonstrating their potential as efficient bioreactors for MF [61].

## 4 Exploring the potential of plants in biopharmaceutical manufacturing

Biopharmaceuticals offer several benefits. For instance, they rarely result in the negative impacts connected with traditional small-molecule therapies since they selectively target molecules. Biopharmaceuticals also show great selectivity and activity in comparison to conventional therapeutics [63]. Recombinant human insulin made from bacteria was the earliest pharmaceutical product to be developed for the market in 1982 [64]. Following the expression of interferon, mAb, serum albumin, and hGH fusion protein, the potential of plants for pharmaceutical manufacture was quickly demonstrated. Since then, there have been several examples of pharmaceuticals manufactured from plants, which are listed under the three major therapeutic groups: Some of the most obvious examples are vaccines, antibodies, and other medications.

### 4.1 Revolutionizing healthcare: transgenic plant vaccines

The discovery of vaccines was a significant accomplishment of the 19th century, and the smallpox vaccine was the first to be developed. The use of vaccinations helped to stop the outspread of epidemic diseases like tetanus, diphtheria, rubella, mumps, polio, hepatitis, and measles [65]. The health of people and animals is now seriously threatened by new infectious diseases. However, using currently available vaccinations is a successful way of stopping the spread of these diseases. Vaccines have evolved from bacterial vaccines to subunit, RNA, and DNA vaccines with the growth of biotechnology, yet these products are challenging to market due to the high cost of production. Most vaccines that are now available on the market are expensive because they require fermenters, extensive production procedures, and expensive technology for purification. Additionally, costs for the formulation, cold storage, transportation, and sterile delivery are incurred [66]. On the other hand, plants offer a cost-effective platform for the development of inexpensive vaccinations. Transgenic plant-derived vaccines have several benefits over conventional injectable vaccines, along with a wide spectrum of potential expressed epitopes, low cost, massive cultivation of biomass, and ease of transportation and preservation [67].

Genetically altered plant vaccine research has advanced for more than 20 years. There have been several effects in the studies of plant-derived medicines and vaccines for people and animals [32] since the development of hGH in transgenic tobacco. Purifying and isolating the r-proteins generated in plant tissue enables the progress of transgenic plant vaccines. The transgenic antigen purified and isolated from plants can produce particular antibodies in animals [68]. As an alternative, the r-protein could be produced in plants' edible parts without further processing for ingestion [69]. The use of edible plants that express heterologous antigens eliminates the need for the recombinant product to be extracted and purified, and the biomolecule is guarded by an outer membrane for increased and sustained activity on mucosal surfaces [70].

Transgenic potato tubers and lettuce leaves that expressed the Hepatitis B virus (HBV) surface antigen (HBsAg) were the earliest plant-produced vaccine candidates to undergo clinical trials [71]. Recombinant vaccines can be developed using a tobacco plant efficiently and economically. Influenza (Flu) vaccine obtained from the *Nicotiana benthamiana* was transfected with a weakened plant viral vector that expressed influenza haemagglutinin (HAs) genes to produce virus-like particles (VLP) [72]. Medicago Inc. created a highly engineered VP form of vaccine candidate against influenza that contains a plant leader peptide with a C-terminal heterologous transmembrane domine. The vaccine was approved by the FDA after clinical trials verified its efficacy and safety [73]. The heavy chain-GP1 fusion protein (H2GP1) was co-expressed with the light chain (K3) to create an Ebola immune complex (EIC). An EIC was created using a geminiviral replicon system in *N. benthamiana*. Humanized 6D8 IgG mAb was bound to the C-terminus of the heavy chain (H-chain) of the Ebola glycoprotein, which fused exclusively to a GP1 linear epitope. These findings contribute to an EIC expressed in plants that had good potential as a human vaccination [74]. The development of recombinant antigen (r-antigen) for foot and mouth disease virus (FMDV) by the *FMDV VP1* gene constructed with the chloroplast expression vector and introduced to tobacco by gene gun method was already reported [75]. Dengue virus E glycoprotein domain III (EIII) is immunogenic and able to trigger the production of antibodies that can neutralize it. A DNA region coding EIII was transferred into *Nicotiana tabacum* plant cells. The efficacy of adopting plant-based vaccines to guard against dengue virus infection is demonstrated by the development of domain III of EIII in transgenic tobacco [76]. Pollet et al. [77] investigated that the receptor binding domain (RBD) gene integrated into *N. benthamiana* produced the spick (S) protein of SARS-CoV-2. Tobacco plants have been genetically altered to produce the extracellular region of the HAs proteins from the H5N1 avian influenza virus opening the way to cheaper and cost-effective vaccine development options [78].

MF has played a minimal role in preventing MERS and SARS, from reaching a critical pandemic level, large-scale response is in the offing. However, the SARS-CoV S-protein's ectodomain was produced in tomatoes, tobacco, and lettuce and was often shown to be immunogenic [79]. Additionally, *N. benthamiana* was employed to transiently express the SARS-CoV nucleocapsid and membrane protein, giving further proof of immunogenicity [80]. Recently, Ghorbani et al. [81] experimented with an immunoinformatic technique to predict the VLP of SARS-CoV-2 epitopes that were exposed to HBcAg to produce a vaccine against SARS-CoV. The chloroplasts of transplastomic tobacco plants as well as the cytosol of transgenics as evidence suggesting the potential development of an oral vaccination against the SARS-CoV. The viral antigen expressed in transformed plants effectively responded to serum and mucosal immunity as well as used for serological antibody detection tests [82]. Charland et al. [83] produced a potential coronavirus VLP (CoVLP) vaccine made in plants that exhibit the SARS-CoV-2 S-protein and coupled with AS03 was taken into use in the preliminary evaluation of phase 2 of Phase 2/3 clinical trials. Shanmugaraj et al. [84] demonstrated that the Baiya SARS-CoV-2 Vax 1 subunit proteins, which were created via the fusion of the SARS-CoV-2 RBD to the human IgG1 Fc domain (RBD-Fc), developed in *N. benthamiana*, and coupled with alum, created strong immune responses in both cynomolgus monkeys and mice [85]. Margolin et al. [86] investigated how to make a soluble plant-derived SARS-CoV-2 spike that included the ectopic glycoprotein, this was transiently expressed in *N. benthamiana*, which greatly improved the accumulation of the glycoprotein (GP). Affordable and reliable manufacturing platforms will increase the availability of vaccinations in countries with limited resources and a large volume of vaccine manufacture is urgently needed. Plant species that provide such platforms to efficiently produce vaccines were described in several research [87]. In immunized hamsters, the *N. benthamiana* produced antigen developed neutralizing antibodies against both the identical Wuhan and heterologous SARS-CoV-2 delta variants, however, titres were reduced compared to those promoted by the comparator mammalian antigen [87]. Health Canada authorized Covifenz, the first SARS-CoV-2 vaccine made from plants (Tobacco, potato, and *N. benthamiana*), in 2022 [88].

The transgenic maize with the rabies virus *G* gene expressed in five putative lines and administrated various doses of G protein through oral delivery in sheep responded productively [89]. Zhang et al. [90] developed an effective oral vaccine, that enhanced the expression of recombinant Norwalk virus (rNV) VLPs in transient tomato plants. It produced elevated levels of (approximately 26.6 μg/g of dry fruit) rNV proteins than previously reported rNV production in potato/tobacco plants [91]. Similarly, Norwalk virus (NV) VLP was obtained from transfected *N. benthamiana* immunized against the NV [92]. The pBRSAg expression vector constructed with the HBsAg gene was transfected into tomato. The transformed tomato plants produced surface antigen for HBV and this was the first reported plant vaccine for HBV [93]. The most frequent cause of extreme diarrhea in juvenile people is rotaviruses (RV), which are thought to account for close to one million fatalities per year globally in past decades. Wu et al. [94] the study revealed that even after the incorporation of the *G1 VP7* gene cloned with PB1131 expression vector was transferred into the potato genome, RV glycoprotein VP7 might still maintain its ability to prevent RV is a challenge. IgG levels were induced by the VP7 DNA vaccine in mice that can't protect from RV. However, mice that were vaccinated with the transgenic tubers produced mucosal IgA and serum IgG antibodies that were specific for VP7. RV might be neutralized by IgA, according to neutralizing tests. These findings suggest that edible RV vaccines might be produced and delivered by plants. Transgenic potato tubers and lettuce leaves expressing the HBsAg [95], maize and potato transgenic lines produced the enterotoxigenic *E. Coli* labile toxin B- subunit [96], transgenic potatoes expressing the NV capsid protein (CP) [97], and virus-introduced spinach producing rabies virus GP [98] were the first vaccine candidates produced from plants to enter clinical progress. The need for a Zika viral (ZIKV) vaccine is urgent given the current global outbreak of the virus and its associated neurological problems in adults, microcephaly in fetuses and infants, and other birth defects. As a result, Yang et al. [99] created a vaccine subunit based on the ZIKV envelope E (zE) protein and studied the immunogenicity of the vaccine in mice. zE quickly increased in *N. benthamiana* plant leaves because of transient expression. Biochemical testing demonstrated a range of mAbs that detect different zE conformational epitopes specifically bound to plant-produced zE (PzE), according to the results of the testing. Given that two dosages of PzE caused potent zE-specific antibodies and cellular immunological activities in mice, it has been shown that PzE is highly immunogenic.

Ward et al. [100] provided results from two phase III efficacy trials of a plant-produced recombinant quadrivalent virus-like particle (QVLP) influenza vaccine, one in adults aged 18–64 years, and the other in older individuals aged 65 years and over. With the use of this platform, which relies on the transient expression of proteins in the plant *N. benthamiana*, VLPs containing HAs protein trimers may be produced, which can then be joined to create a QVLP vaccination [101]. In both pre-clinical and clinical tests, it has been found that influenza HAs-containing plant-derived VLP vaccines elicit potent CD4^+^ T and humoral responses. These insights could contribute to an explanation of the vast and cross-reactive immune responses induced by plant-based vaccinations [102]. Jeshvaghani et al. [103] examined the oral immunogenicity of a synthetic r-protein (SICL) including LtB, CfaB, StxB, and Intimin. Serum IgA and fecal IgG antibody responses were produced after the oral vaccination of mice. These findings showed that the fusion r-protein SICL in canola transgenic seed functions as a potent immunogen, inducing both mucosal and systemic immunogenicity as well as defense against ETEC and EHEC toxicity. A malarial candidate vaccine produced through plants (*N. benthamiana*) based on Pfs25 has been created as a chimeric non-enveloped VLP that combines Pfs25 with the CP of the alfalfa mosaic virus. However, the vaccination did produce dose-dependent Pfs25-specific IgG in vaccinated individuals [104]. The cytoskeletal VP1 of the FMDV expressed in transgenic *Stylosanthes guianensis* cv. Reyan II has been used to design a new oral vaccination method for the protection of FMDV [105].

Plastid transformation has successfully been used to express vaccination antigens. Yácono et al. [106] investigated chloroplast-mediated production of the anti-GRA4 for *Toxoplasma gondii* in tobacco plants and assessed the cellular and humoral response and level of protection following oral delivery of chlGRA4 in a mouse model. Mice that were administered with an oral dose of the chlGRA4 vaccine had 59 % fewer brain cysts than control mice. A mucosal immunity of the chlGRA4 vaccine was described by the development of specific IgA, and a systemic immunological response was characterized by the creation of GRA4-specific serum antibodies and the production of IL-4 and 10, and IFN-c by splenocytes. These findings show that oral delivery of chlGRA4 encourages the Th1/Th2 responses, which regulate Toxoplasma infection. In transgenic *Solanum lycopersicum* hairy roots, the expression of an r-protein, which comprised the ricin toxin B chain and the rabies glycoprotein (rgp-rtxB) fusion antigen, was controlled by a constitutive CaMV35S promoter. Following intra-mucosal immunization, the partially refined RGP-RTXB chimeric protein successfully induced an immune response in BALB/c mice [107]. A tobacco mosaic viral transient expression system was used to create a plant-optimized gene expressing Narita 104 virus (NaV) CP in *N. benthamiana*. At 4 days following infection, NaVCP had accumulated to a maximum of around 0.3 mg/g of fresh leaf weight [108]. The parallel expression of four different proteins in different concentrations was used to prevent the Bluetongue virus (BTV), which has a negative influence on the livestock trade due to its high death rates in ruminants. Thuenemann et al. [109] revealed that VLPs generated and constructed in *N. benthamiana* utilizing the cowpea mosaic virus-based HyperTrans (CPMV-HT) and related pEAQ transient expression system induced a potent antibody response in sheep.

There are multiple existing clinical studies employing purified antigens, produced rapidly in vaccine particles from tobacco plants. For instance, a phase II clinical investigation taken on a flu QVLP created from plants (*N. benthamiana*) was accomplished, and it was newly reported that phase III clinical research is underway [101]. The current status of research on plant-based vaccines against viruses is quite promising. Recent advancements have demonstrated the efficacy of PMF in producing viral vaccines and therapies. Notable progress includes the successful use of plant-made antigens and mAbs for treating animal and human viral diseases. HAC1, an *N. benthamiana* -produced recombinant HAs flu vaccine created by Fraunhofer USA Center for Molecular Biotechnology (FhCMB) for the treatment of a disease caused by a new H1N1, phase 1 trial was evaluated for safety and immunogenicity first-in-human. In less than a month, FhCMB designed, developed, and manufactured HAC1, and then scaled it up for current Good Manufacturing Practices in FhCMB's pilot plant [110]. Also plant recombinant HAs protein was examined in phase I human trial against H5N1 influenza stain [111]. The 6-g dose of MucoRice-cholera toxin (CTB) vaccination produced cross-reactive antigen-specific antibodies against the B subunit of CTB and *E. coli* heat-labile enterotoxin without causing significant side effects, according to a phase I study presented by Yuki et al. [112]. The study presented a phase I trial that was double-blind, randomized, and placebo-controlled, demonstrating that oral MucoRice-CTB induces neutralizing antibodies against CTB regardless of ethnicity. Despite advancements in subunit vaccines through transient expression techniques, no plant-derived recombinant vaccine has received approval for human use to date [112]. The successful expression of plant-based vaccine candidates is presented in Table 2. The lack of plant-based vaccines can be attributed to several key hurdles that need to be addressed. Firstly, navigating the complex regulatory landscape poses a significant challenge, as these vaccines must meet stringent criteria for safety, efficacy, and environmental impact to gain approval. Additionally, the commercialization process of plant-made vaccines involves a multitude of steps and collaborations across different groups, requiring careful consideration of factors such as business planning, financing, and regulatory compliance before significant investments are made. There is also a notable knowledge gap between the laboratory research phase and the commercialization phase, highlighting the need for a more seamless transition that encompasses aspects like business planning, financing, and regulatory approval. Cost considerations and low adoption rates among farmers due to the expense and handling requirements of traditional vaccines further hinder the uptake of plant-based alternatives. Environmental concerns surrounding the safety of vaccine-producing plants intended for food or feed crops also present challenges, necessitating strict oversight and production conditions to ensure regulatory compliance. By addressing these hurdles, the development and adoption of plant-based vaccines can be facilitated, offering a promising avenue for more efficient and cost-effective veterinary solutions [113]. To overcome the key hurdles hindering the development and adoption of plant-based vaccines, several strategies can be implemented in the near future. Collaboration with regulatory authorities to streamline approval processes and provide comprehensive data on safety and efficacy is essential. Enhanced partnerships between research institutions, industry players, and regulatory bodies can facilitate knowledge sharing and efficient progression from research to commercialization. Increased investment in research and development of plant-based vaccine technology is crucial to address knowledge gaps and enhance production efficiency [114]. Exploring cost reduction strategies, such as optimizing manufacturing processes and scaling up production, can make plant-based vaccines more economically viable. Educational campaigns to raise awareness among farmers and stakeholders about the benefits and safety of these vaccines can help increase adoption rates. Implementing stringent environmental monitoring and containment measures for vaccine-producing plants is necessary to address safety concerns. Advocating for supportive policies and incentives from government agencies can create a conducive regulatory environment for the development and adoption of plant-based vaccines in the veterinary industry [115]. By systematically implementing these strategies, the future prospects for plant-based vaccines in veterinary medicine can be significantly enhanced, leading to improved animal health, cost-effective solutions, and sustainable agricultural practices.

**Table 2:** Vaccine candidates expressed in plant system.

Disease/Pathogen	Plant	Expression system	Antigen/Antibody	Reference
Anthrax	Tobacco	Biolistic Stable^a^	PA	[197]
Avian chlamydiosis	Rice	Agrobacterium	MOMP	[198]
Avian influenza	*N. benthamiana*, Alfalfa, soybean, Tobacco, Duckweed		HAs (H7, H5, H6)	[199]
*Bacillus anthracis*	Tobacco	Agrobacterium	PA (dIV)	[200]
*B. anthracis*	*N. benthamiana*	Transient	PA	[201]
Bluetongue virus	*Nicotiana benthamiana*	Agrobacterium Transient	Bluetongue-VLP	[109]
Bovine herpes virus	*N. benthamiana*	Agrobacterium^b^	Glycoprotein D	[202]
BVDV	Alfalfa	Agrobacterium^b^	Structural protein E2	[203]
*Brugia malayi*	Tobacco	Agrobacterium	Bm ALT-2	[204]
Cholera	Potato	Agrobacterium	CTB	[205]
Cholera	Tomato	Agrobacterium	CTB	[206]
Cholera	Rice, Maize	Particle Bombardment	CTB	[207,208]
Cottontail rabbit Papillomavirus	Tobacco	Agrobacterium^b^	L1-CP	[209]
CCHFV	Tobacco	Stable, Transient expression	G1/G2	[210]
Cysticercosis	Papaya	Particle Bombardment	KETc1, KETc12, KETc7	[211]
Diarrhea	*Medicago sativa*		E2	[203]
*E. coli*	Potato	Agrobacterium	Enterotoxin B subunit	[212,213]
*E. coli*	Maize	Agrobacterium	LT-B	[214]
*E. coli*	Rice	Microprojectile Bombardment	Enterotoxin B subunit	[215]
*E. coli*	Lettuce	Agrobacterium	LT-B	[216]
*E. coli*	Soyabean		LT-B	[217]
*E. coli*	Rapeseed		SICL (StxB, Intimin, CfaB, LtB)	[103]
*E. coli*	Corn	Agrobacterium	LT-B	[96]
*E. coli*	Arabidopsis	Agrobacterium	LT-B	[218]
*E. coli*	Alfalfa	Chloroplast^a^	FaeG	[219]
*E. coli*	Carrot	Agrobacterium^b^	LT-B	[220]
Ebola	*N. benthamiana*	Transient expression	GP1	[74]
FMDV	*Stylosanthes guianensis*	Agrobacterium^b^	VP1	[105]
H1N1 (Influenza A)	*N. benthamiana*		HAC1	[110]
H3N2	Maize	Agrobacterium	Nucleoprotein	[221]
H5N1	*N. benthamiana*	Transient expression	HAs protein	[111]
H5N1	*N. benthamiana*	Agrobacterium Transient	HAs protein	[222]
H5N1 & H1N1	*N. benthamiana*	Agrobacterium Transient	HAs protein	[223]
H7N7	*N. benthamiana*	Agrobacterium Transient	HAs protein	[224]
HBV	Tobacco	Agrobacterium^b^	HBsAg	[225]
HBV	Banana cv. Rasthali	Agrobacterium	HBsAg	[226]
HBV	Tobacco	Agrobacterium	HBsAg	[227]
HBV	Rice	Agrobacterium	HBsAg	[228]
HBV	Tomato	Electroporation, Agrobacterium	HBsAg	[229,230]
HBV	Potato		HBsAg	[231]
HBV	Lettuce	Agrobacterium	S-HBsAg	[232]
HBV	Maize		HBsAg	[233]
HBV	Potato	Agrobacterium^b^	HBsAg	[234]
HBV	*N. benthamiana*	Agrobacterium^b^	HBsAg	[235]
*Helicobacter pylori*	Carrot	Agrobacterium	UreB (urease B)	[236]
HIV	Arabidopsis, carrot		HIV-1 p24	[237]
HIV	Lettuce	Agrobacterium	C4(V3)6	[238]
HIV	Tobacco	Biolistic transformation^a^	anti-C4V3	[239]
HIV type 1	Tobacco	Biolistic Stable^a^	Pr55^gag^ Polyprotein	[240,241]
HIV-1	Tobacco		p24-Nef	[242]
HPV	Tomato		HPVL1-E6/E7	[243]
HPV	Tobacco	Agrobacterium^b^	L1-CP	[244]
HPV	Tobacco		HPV16-L1	[245]
IBV	Potato	Agrobacterium^b^	S protein	[246]
IBDV	Rice	Agrobacterium^b^	VP2	[247]
Influenza	*N. benthamiana*	Agrobacterium Transient	HAs protein	[248]
Influenza		Agrobacterium	QVLP	[100,249]
Japanese encephalitis virus	Rice	Agrobacterium	E protein	[250]
Japanese encephalitis virus	Nipponbare	Agrobacterium^b^	Envelope protein (E)	[250]
Malaria	*N. benthamiana*		Pfs25-VLP	[104]
*Mycobacterium tuberculosis*	Carrot		ESAT6, CFP10	[251]
*Mycobacterium tuberculosis*	Tobacco, Lettuce	Chloroplast expression^a^	ESAT6, Mtb72F, Mtb32	[252]
Narita 104 virus	*Nicotiana benthamiana*	Agrobacterium Transient	NaV-VLP	[108]
NDV	*N. benthamiana*		HN protein	[253]
NDV	Maize	Biolistic Stable^a^	Fusion (F) protein	[254]
NDV	Tobacco	Agrobacterium	GST–HN	[255]
Norwalk virus	Tobacco, Potato	Agrobacterium	rNV-CP	[91]
Norwalk virus	*N. benthamiana*	Agrobacterium^b^	CP	[256]
Norwalk virus	Tomato		rNV	[90]
Norwalk virus	*N. benthamiana*	Agrobacterium	rNV	[256]
*Plasmodium falciparum*	Tobacco/Lettuce		AMA1^a^	[257]
*P. falciparum*	Tobacco/Lettuce		MSP1^a^	[257]
Poliovirus	Tobacco		VP 1	[258]
PRRSV	Arabidopsis	Agrobacterium^b^	Glycoprotein	[68]
RHDV	Tobacco	Agrobacterium^b^	VP60	[259]
Rabies	Maize	Biolistics	G protein	[89]
Rabies	Tomato		RGP–RTB	[107]
Rabies	Tobacco	Agroinfiltration	N protein	[260]
Rinderpest virus	Peanut	Agrobacterium^b^	HAs protein	[261]
Rotavirus	*M. sativa*		VP6	[262]
SARS-CoV	Tomato, Tobacco	Agrobacterium	S1 protein	[82,263]
SARS-CoV	*N. benthamiana*	Agrobacterium	SARS-CoV nucleo-capsid	[264]
SARS-CoV, SARS-CoV-2	Cowpea		VLPs	[265]
SARS-CoV-2	*N. benthamiana*		S Protein	[266,267]
SARS-CoV-2	Tobacco	Transient	S glycoprotein	[268]
SARS-CoV-2	*N. benthamiana*	Transient	RBD-Fc	[84]
Tetanus/*Clostridium tetani*	Tobacco	Chloroplast expression^a^	TetC	[89]
*T. gondii*	Tobacco	Biolistic transformation^a^	GRA4 antigen	[106]
*T. gondii*	Tobacco		SAG1	[269]
TGEV	Tobacco	Agrobacterium^b^	S protein	[270]
TGEV	Maize	Agrobacterium^b^	S protein	[271]
*Vibrio cholerae*	Tobacco	Biolistic transformation^a^	CTB-GFP	[272]
*V. cholerae*	Rice		CTB	[112]
*V. cholerae, E. coli*	Tobacco	a	LTB, CTB	[273]
*Yersinia pestis*	Tomato	Transient	F1-V	[274]
*Y. pestis*			F1-V fusion antigen^a^	[275]
Zika virus	*N. benthamiana*	Transient expression	zE	[99]

a Chloroplast transformation.

b Stable Transformation.

### 4.2 Plantibodies: the future of antibody therapeutics

The adaptive immune system of animals creates glycoproteins called antibodies. They have high specificity and affinity for recognizing and binding specific target antigens, which enables them to eliminate pathogens and other foreign substances from the body [116]. There are five kinds of immunoglobulins produced by mammals, each having a unique Fc (fragment crystallizable) region and corresponding effector function (IgA, IgD, IgE, IgG, and IgM). IgG is the predominant serum antibody in mammals [117]. Immunotherapy has tremendous results for diseases that were previously thought to be incurable with the effective therapeutic mAbs now being developed [118]. The mAbs are the primary type of biopharmaceutical products at present. However, they are generally utilized to treat certain diseases that each have relatively low occurrences [119]. Around 1990, the term "plantibody" was introduced to describe plants as possible hosts for producing antibodies [120]. Plants cannot natively produce antibodies, but by adding the relevant immunoglobulin genes, they may be made to do so [121]. Therefore, two genes - one each for the light and H-chains are required for the development of a full-size IgG in plants. While a secretory immunoglobulin A (sIgA) expression needs four genes, one for each of its elements [116]. Plantibodies were produced by the following methods, Conventional method [122]; *In vitro* cell and tissue culture [123]; Breeding and sexual crossing [124]; and Transgenic seeds [125].

*Porphyromonas gingivalis* has a structural subunit Fimbrial protein fimbrillin (FimA) has been recommended as a candidate vaccine to reduce periodontitis induced by *P. gingivalis* [126]. Choi et al. [126] investigated the biological functions of the FimA-specific mAb created in the plant (anti-FimA plantibody) suppressed *in vivo/in vitro*. *P. gingivalis* infections of periodontal ligament cells. In comparison to the death of bacterial cells with extraneous IgG, mouse macrophages opsonized with the anti-FimA mAb dramatically improved the intracellular killing of *P. gingivalis*. Protein microbicides against HIV can aid in preventing infection if they are administered in high and frequent doses leading to high prices [126]. Plants are useful as manufacturing platforms because they provide secure, affordable, and scalable substitutions, which would make it possible to create therapeutic proteins on a budget and in large quantities [127]. Wide-ranging HIV neutralization activity is observed in human mAb 2G12. The 2G12 production has widely been delineated in various plants [128]. Vamvaka et al. [127] described the production of transgenic rice plants that express the HIV-neutralizing mAb 2G12 in the endosperm, The H-chain was largely glycosylated for an antibody whereas the heavy and light chains combined to form effective antibodies with stronger HIV-neutralizing activity was observed. Recently, a transient expression approach was used in *N. benthamiana* to produce a novel anti-HIV-1 bispecific bNAb-lectin fusion protein [129], Macaques [130], and rice endosperm [131]. The system's potential for molecular farming was evaluated by developing doubled haploid transgenic barley that was designed to produce r-protein around 160 μg per g grain of the anti-HIV-1 mAb 2G12. In accordance with its vacuolar localization, the r-protein was observed close to the edges of protein bodies as a combination of different N-glycans [132].

Recombinant mucosal antibodies are promising molecular targets for the design of next-gen biotherapeutics for the prevention of several serious diseases through passive immunizations and the treatment of mucosal antibody deficits in patients [133]. IgA is a potential oral passive immunization antibody against mucosal bacteria like *E. coli* that produces the Shiga toxin [134]. Nakanishi et al. [134] developed cDNA that encodes an anti-Stx1 antibody, and all the H-chain constant regions and variable regions were taken from a mouse IgA mAb. They used a plant expression system to generate the cDNA to produce enough IgA at a reasonable cost in consideration of oral delivery. The effective expression of sIgA and sIgM in diverse antibody formats in a diverse of plants, including tobacco, maize, tomato, and its close relatives *N. benthamiana*, and *A. thaliana*, demonstrates that plant cells are capable of producing high-quality mucosal antibodies [133]. Loos et al. [135] presented a comparison of two mAbs (HA78 and 2G12 against Hepatitis A and HIV respectively), expressed in wild-type (WT) seeds of Arabidopsis plants and glycosylation mutants missing plant-specific N-glycan residues. The epitope specificity or neutralization activity of HIV-neutralizing antibody 4E10 and the human tumor-specific antibody M12 was analyzed as a potential reason for the yields developed in *N. benthamiana* [136]. Three anti-Ebola chimeric mAbs were produced in *N. benthamiana*. This pilot experiment results showed the successful postexposure efficiency of mAb produced from *N. benthamiana* [137]. Additionally, the development and testing of vaccines like ZMapp for Ebolavirus infection showcase the potential of plant-based approaches in combating viral infections [138]. ZMapp is composed of three distinct mAbs, each specifically designed to target the Ebola virus. The ZMapp vector contains genes (MB-003, 1H3, and 13C6) that encode these three antibodies. The Ebola virus is effectively neutralized by a combination of three antibodies that are generated in tobacco plants utilizing a plant-based expression approach [139,140]. The study assessed the viability of using a plant expression approach to create the B38 and H4 human anti-SARS-CoV-2 mAbs quickly which were recently found to be clinically useful. By employing a plant expression geminiviral vector to selectively co-express the light and heavy-chain sequences of both antibodies, it was able to rapidly accumulate formed mAbs in *N. benthamiana* leaves four days after infiltration [141].

The development of transgenic tomato plants that express recombinant human IgA, targeted against the VP8 peptide of the SA11 RV strain. The fruit of the modified plants contained 3.6 % of the total soluble protein as IgA. In an *in vitro* viral neutralizing study, negligibly processed fruit-derived products fit for oral consumption showed anti-VP8 binding activity and substantially suppressed viral infection [142]. Utilization of recombinant sIgA complexes potentially increases antibody-based passive immunomodulatory strategies for treating a variety of mucosal infections [143]. The broad neutralizing anti-HIV mAb 2G12 was expressed, purified, and characterized using transgenic tobacco and transient *N. benthamiana*. Heterodecameric complexes were generated and accumulated in vacuoles and other intracellular spaces within leaf tissue. The antibody yield in transgenic tobacco reached 15.2 mg/g of leaf fresh mass (LFM) and increased to 25 mg/g LFM with the transient system. Transgenic tobacco demonstrated superior sIgA complex assembly. The protein L purified antibody exhibited precise binding to HIV gp140 and effectively neutralized HIV 2 and 3 isolates [144]. Compared to animal-produced antibodies, these are less expensive, safer to use, and simpler to handle [145]. The production cost of 1 g mAb in transgenic plants is anticipated to be around 0.10 USD [119], which is far less than the usual cost of 53–106 USD per g of CHO cell production [145]. Safety issues and handling of antibodies produced in plants are critical considerations in the development of plant-based pharmaceuticals. A comprehensive risk assessment for viral safety is conducted, evaluating factors like raw materials, operators, process aids, equipment/utilities, and environmental conditions. Quality control measures are implemented throughout the production process, including the use of certified raw materials, operations in controlled environments, and virus removal steps like nanofiltration [146]. In the first-in-human phase I clinical trial, the primary focus is on evaluating the safety and reactogenicity of the plant-derived mAbs when administered intravaginally. Regulatory compliance is paramount, with the project following guidelines to ensure adherence to standards. Future research is needed to further characterize the pharmacokinetic properties of plant-derived antibodies and monitor their safety and efficacy. These efforts underscore the importance of stringent safety assessments, quality control, and regulatory compliance in the production and handling of antibodies derived from plants [128].

An alphavirus transmitted through mosquitoes named the chikungunya virus (CHIKV) can infect people and lead to chronic, severe arthritis. There aren't any approved medicines or vaccines for treating CHIKV infections in humans at the moment. The potential for treating CHIKV in an animal model utilizing anti-CHIKV mAbs generated in glycoengineered (ΔXFT) and Wild type (WT) *N. benthamiana* was examined by Hurtado et al. [147]. Strong *in vitro* neutralizing activity against CHIKV was shown for both ΔXFT and WT plant-produced mAbs. Notably, both mAb glycoforms exhibited *in vivo* effectiveness in test models, with the mAbs produced by the ΔXFT exhibiting slightly greater efficacy [147]. Politch et al. [148] performed a phase I trial in a multipurpose preventive technology (MPT) product called MB66 film, containing mAbs against HSV-1, 2, and HIV-1. *N. benthamiana* was used to produce the mAbs by the transient system. According to the observations of this MB66 Phase I clinical trial, both single and repeated vaginal applications of a mAbs film against various HIV and HSV, which had been made using a quick, economical plant-based system, were well tolerated and safe. The film also delivered antibody concentrations in vaginal secretions that were able to neutralize HSV-2 and HIV-1 for a minimum of 24 h succeeding film application. As a result, plantibodies are now widely employed in the medical, veterinary, and other fields of commercial activity. They are utilized to treat malignancies, inflammatory diseases, and immunological disorders [148]. Clinical studies are now being conducted on many plant-produced antibodies. Reported plantibody production in plant systems is presented in Table 3.

**Table 3:** MF system for antibody production in plants.

Plantibody	Antigen	Host	Mechanism	Reference
anti-FimA	FimA	Rice cell suspension	Passive immunization against *P. gingivalis*	[126]
B38 and H4	anti-SARS-CoV-2	*N. benthamiana*	Neutralization activity	[141]
IgA	anti-*streptococcus mutants*	Tobacco		[276]
IgG1		Soybean, rice	Herpes simplex virus	[277]
IgG		Corn	Respiratory Syncytial Virus	[278]
mAb 4E10	4E10	*N. benthamiana*	HIV-neutralizing antibody	[136]
mAb M12	Tumour specific M12	*N. benthamiana*	human tumor-specific antibody	[136,279]
mAbs	P2G12	Tobacco	HIV neutralizing	[128]
mAbs	HA78	Arabidopsis	Hepatitis A virus	[135]
mAbs	2G12	Arabidopsis	HIV-neutralizing antibody	[135]
mAbs	2G12, 2F5	Tobacco, Maize, Rice	HIV-specific antibodies	[127,128,131,280,281]
mAbs	anti-HIV-1 2G12	Barley	HIV-neutralizing antibody	[132]
scFv	CLas proteins InvA and TolC	Carrizo	Greening Disease	[282]
Guy's 13	Streptococcal antigen I or II	Tobacco	Dental caries	[283]
ScFvT 84.66	Carcinoembryonic antigen	Wheat, Pea Rice	Cancer treatment	[284,285]
T84.66	Carcinoembryonic antigen	Tobacco	Cancer treatment	[286]
CO17-1A	Surface antigen	*N. benthamiana*	Colon cancer	[4]
Anti-HSV-2	Herpes simplex virus 2	Soybean	Viral neutralizing	[277]
anti-HBsAg	HBV	Tobacco	sAg antibody	[287]
anti-Stx1		Arabidopsis	mAb for *E. coli*	[134]
BR55-2 mAb	Anti-Lewis Y	Tobacco		[288]
cT84.66, scFvT84.66	Anti-cancer	Tobacco		[286]
H10 mAb	Anti-TNC	Tobacco		[289]
hIgA_2A1	anti-VP8*	Tomato	Retrovirus neutralizing	[142]
IgG		Tobacco	Viral neutralizing	[290]
mAb	anti-CHIKV	*N. benthamiana*	Neutralizing	[147]
mAb		*N. benthamiana*	HIV and HSV neutralization activity	[148]
mAb	PA	*N. benthamiana*	neutralize toxin activity of *B. anthracis*	[291]
mAb	Anti-PA	*N. benthamiana*	Anthrax neutralizing	[291]
mAb BR55-2	Anti-Lewis Y	Tobacco	Cancer neutralizes	[288]
P2G12 SIgA	anti-HIV	*N. benthamiana*		[144]
R12 mAb		Tobacco	Rabies treatment	[292]
Rabies virus glycoprotein		Tomato	Rabies treatment	[293]
RIG		Tobacco	Neutralizing	[294]
scFv	anti-BoNT/A	Tobacco	Immunization against *Clostridium botulinum*	[295]
scFv	Anti- *Salmonella*	Tobacco	Immunization against *Salmonella enterica*	[296]
scFv	Anti-BoNT/A	Tobacco	Immunization against Botulism	[295]
scFv	anti-LPS		Immunization against Salmonella	[296]
scFv, IgG1, diabody	anti-CEA	Rice, wheat, pea, tomato	Neutralize carcinoembryonic antigen	[297]
scFvT84.66	Ant-cancer	Oryza sativa, Wheat	Tumour neutralizing	[284,285]
sIgA	anti-VP8*	*N. benthamiana*	Retrovirus neutralizing	[298]
SlgA		Corn	Herpes simplex virus	[299]
CaroRX		Tobacco	Immunization against *Streptococcus mutans*	[300]
VHH-IgG, VHH-IgA	anti-ETEC	Arabidopsis		[301]
VHH-Fc		Arabidopsis, *N. benthamiana*		[302]
ZMapp		*N. benthamiana*	Ebola neutralization	[303]

### 4.3 MF platforms for protein and enzyme production in plants

MF can be used by plants to produce r-protein products. r-proteins play essential roles in various aspects of our lives, contributing to advancements in healthcare, agriculture, industry, and research. Recombinant enzymes are employed as great-quality proteins for research, therapeutic, and diagnostic purposes, as well as in the chemical and food industries [149]. Recombinant pharmaceutical proteins and industrial enzymes are now increasingly being produced in plants as factories [150]. r-proteins can also be released into the culture media, enabling recovery and ensuing purification since cells retain the majority of the contaminated proteins and can be eliminated from the culture media [151]. In specific, the MF of cell-wall-deconstructing enzymes like hemicellulases, ligninases, xylanases, and cellulases has enormous potential for the biofuel sector concerning the manufacturing of cellulosic ethanol [152]. The hundreds of various proteins in plants were successfully produced including medicinal proteins such as hormones, antibodies, vaccines, and enzymes, along with proteins for cosmetic, research, and diagnostic applications [149]. The earlier studies on MF reveal a diverse landscape characterized by challenges in establishing standardized processes due to the wide range of expression strategies and production systems. Despite the success of traditional biotechnology platforms, versatility is offered by molecular farming for different products and applications. The increasing interest in the downstream processing of plant-made pharmaceuticals is evidenced by a rise in publications and expanding manufacturing capacities [71]. The broad spectrum of species and production techniques working in the plant-based expression of proteins is a unique property [149]. Accordingly, the MF community has started emphasizing a few platforms, with tobacco and its related species *N. benthamiana* popularly [71]. Animals were commonly used in further studies with therapeutic proteins. For example, the West Nile virus (WNV) envelope protein domain III created in *N. benthamiana* prevented mice from fatal WNV infections [153], while the transiently developed hepatitis C virus E1E2 heterodimer in lettuce induced immunological activity in mice [154].

These proteins are produced in plants, which has benefits such as authentic plant allergen presentation for allergy detection [155] and lack of contamination with animal-derived components or bacterial endotoxins, which are required for producing GF and cytokines as they are used in food production or animal cell culture [156]. Refined protein is employed as a bioactive component in cosmetic products [157]. Therefore, there has been an abundance of interest in transgenic plants as the bioreactors of a new generation because of their benefits, such as the reliability of recombinant molecules (vaccines, antibodies, r-proteins, GF, enzymes, etc.), and the large-scale execution achieved at low cost [62]. Transgenic plants have emerged as a potential replacement for conventional bioproduction approaches, such as cultured mammalian cells, yeast, and bacteria, for the development of expensive r-proteins for industrial and medicinal sectors [61]. Cathepsin D inhibitor can serve as the structural stabilizer to maintain the targeted r-proteins in the plant cytosols [158], and this signal has shown to be extremely efficient and cost-effective for producing r-protein. The Israeli company Protalix Biotherapeutics develops taliglucerase alfa, a recombinant isoform of human glucocerebrosidase (GCase), in plant suspension cultures [159]. Another version of the recombinant enzyme (imiglucerase) is produced in CHO cells [160]. Although taliglucerase alfa is the only r-protein produced by plants that have received standard clinical permission for pharmaceutical use in humans, several additional medicine candidates have already advanced to late-stage medical trials. For example, Pegunigalsidase alfa has recently begun phase III trials, a recombinant type of human globotriaosylceramide (Gb3) generated in tobacco as an enzyme substitution medication for Fabry disease [161]. A vaccine produced from plants for seasonal influenza haemagglutinin was transiently produced in *N. benthamiana* to form encapsulated VLPs that mimic WT influenza virions but lack RNA [100]. The driving force behind MF renders its expenses significantly lower than those of traditional farming. One of Franklin and Mayfield's newest production studies interested in the unicellular alga *Chlamydomonas reinhardtii*. The only plant whose transformation was carried out in all of its genome parts (mitochondria, plastid, and nucleus) is *C. reinhardtii* [62].

In recent years, human insulin has become the most widely used recombinant biopharmaceutical with large-scale production, with yearly output averaging over 13 tonnes. Future demand for economical insulin is predicted to significantly grow because of the rising prevalence of diabetes and the development of alternative delivery systems that need greater dosages [162]. Current manufacturing methods will struggle to fulfil this demand due to constraints on production efficiency and capacity. New expression and recovery technique enables the production of biopharmaceuticals from oilseeds at low cost [163]. Human recombinant insulin precursor was produced using this approach and expressed in genetically altered plants [163]. *In vitro,* plant-based insulin can be an enzymatically processed product similar to that of DesB_30_-insulin, which is estimated to accumulate in considerable quantities in transgenic seed (0.13 % of the total protein in the seed) [164]. Numerous other MF products have been created, many of which are presently undergoing human clinical trials as reviewed by Schillberg and Finnern [149]. Examples of MF expression systems and products such as expansion (enzyme), Biofuel production in transgenic *Zea mays* seeds [165], *Daucus carota* produced Elelyso (Enzyme), which is used for Gaucher's disease treatment [166], Artemisinin's derived from *Artemisia annua* and is well established for the medications of malaria [167]. Some examples of PMF-produced proteins and enzymes are mentioned in Table 4.

**Table 4:** Protein and enzymes produced using PMF.

Protein/Enzyme	Plant	Functions	Application	Reference
Aprotinin	*N. benthamiana*	Serine protease inhibitor	Therapeutic	[304]
Artemisinin	Artemisia	Malaria	Therapeutic	[167]
Avidin	Maize	–	Diagnostics	[8]
Basic fibroblast growth factor	Tobacco	–	Clinical	[305]
Dentin matrix protein 1	Tobacco	Osteogenic differentiation	–	[306]
DesB_30_	Arabidopsis	Insulin	Pharmaceutical Protein	[164]
Factor H	*P. patens*	–	Therapeutic	[307]
Glucocerebrosidase	Carrot cell suspension	Gaucher's disease	Therapeutic enzyme	[308,309]
hGH	Pea	–	Therapeutic	[310]
hGH	Tobacco	–	Therapeutic	[311]
hOAT	Lettuce	Antibodies	Therapeutic	[312]
Hirudin	Canola	Thrombin inhibitor	Therapeutic	[293]
Human aprotinin	Maize	Trypsin inhibitor	–	[313]
Human enkephalins	Arabidopsis	Antihyperanalgesic by opiate activity	Therapeutic	[314]
Human erythropoietin	Tobacco	Anaemia	Therapeutic	[314]
Human granulocyte-macrophage colony-stimulating factor	Tobacco	Neutropenia	–	[126]
Human hemoglobin	Tobacco	Blood substitute	Pharmaceutical Protein	[315]
Human hirudin	Rapeseed	Thrombin inhibitor	Therapeutic	[315]
Human homotrimeric collagen	Tobacco	Collagen	Pharmaceutical Protein	[316]
Human interferon	Rice, turnip, Tobacco	Hepatitis C and B treatment	Therapeutic	[314,315]
Human lactoferrin	Potato	Antimicrobial	Therapeutic	[58]
Human lysozyme protein	Rice endosperm	GI infections in infants	Nutraceuticals	[317]
Human protein C	Tobacco	Anticoagulant	Pharmaceutical Protein	[315]
Human serum albumin	Tobacco	–	Pharmaceutical Protein	[318]
Human somatotropin, chloroplast/Nuc	Tobacco	Growth hormone	Pharmaceutical Protein	[319]
Human α-1-antitrypsin	Rice, Maize	Cystic fibrosis, liver disease, and Rice Human -1-antitrypsin	–	[293,313]
Insulin	Arabidopsis	–	Pharmaceutical Protein	[320]
Insulin	Tobacco	–	Diabetes treatment	[321]
Interleukin-2	Tobacco	–	Therapeutic	[322]
Lactoferrin	Rice, Tomato	–	Multifunctional	[323]
Lumbrokinase	Sunflower	anti-thrombosis protein	Therapeutic	[324]
Lysozyme	Rice	–	Numerous	[325]
Osteopontin	*N. benthamiana*	Dental bone regeneration	Therapeutic	[306]
Polyhydroxybuterate	Tobacco	–	Plastic production	[326]
β- Galactosidase	Tobacco, maize	Thermostable cellulolytic enzymes	Industrial	[327]
Zmapp™	*N. benthamiana*	Ebola Zaire virus	Therapeutic	[11]
VEN BETA	Rice	Gastroenteritis	–	
Optibumin	Rice	Loss of albumin	–	
Elelyso	Carrot	Gaucher's disease	Therapeutic	[328]
VEN 100	Rice	Lactoferrin	–	
Canine interferon alpha	Strawberry	–	–	
Gastric lipase	Maize	Cystic fibrosis	Therapeutic	
α-interferon	Rice, Turnip	Anticancer	Pharmaceutical Protein	[293]
α-Galactosidase	Tobacco	Fabry disease	Therapeutic	Adopted and modified [329]
α-interferon	Duckweed	Hepatitis B and C	Therapeutic	
Fibrinolytic drug	Duckweed	Blood clot	Therapeutic	
Insulin	Safflower	Diabetes	Therapeutic	
Apolipoprotein	Safflower	Cardiovascular	Therapeutic	

### 4.4 Secondary metabolites produced by plants' expression system

Tobacco has several distinct benefits across other plant species when it involves protein production with clinical execution. Scientists think that tobacco plays a similar function in research on recombinant proteins as the white mice have in mammalian studies during the past decade. A rare lysosomal storage disorder called Gaucher disease (GD) is caused by mutations in the genes that code for the enzyme -GCase. Several nations have authorized the use of plant-derived taliglucerase alfa as an enzyme substitution therapeutics for the treatment of GD [159]. *Trigonella foenum-graceum* has been found to contain diosgenin, a triterpene with several pharmacological uses. Using the hairy roots (HR) culture platform and the pBI121 expression plasmid, the rate-limiting enzyme in the diosgenin biosynthesis, Δ^24^-reductase, has been overexpressed. Compared to non-transgenic HR, the Δ^24^-reductase expression level was substantially 8.15 times higher in transgenic HR. Additionally, it was increased 25 times when compared to the diosgenin quantity in the "Hamedan" Leaf. The diosgenin aggregation in the transgenic HR was increased three times over the non-transgenic HR [168]. Columbamine O-methyltransferase (CoOMT) is a key catalytic enzyme involved in the biosynthesis of the tetrahydropalmatine path in the alkaloid biosynthetic pathways of Stephania and Rannunculaceae. Its transcription rates were greater in three transgenic tobacco lines from the T0 generation (T0-7, 9, and 20) than in the WT tobacco plants. When compared to tobacco WT, the CoOMT transgenic lines had an estimated 1.09–1.83-fold higher total alkaloid load [169]. Fu et al. [170] investigated the function of *EpOPR1* in the synthesis of these secondary metabolites further using a transgenic HR system. This study revealed that although the chemical characteristics of *EpOPR1* overexpressing HR and empty vector HR are comparable, overexpressing *EpOPR1* line significantly elevated the production of acetyl-shikonin by roughly two-fold. The current studies on the use of plant bioreactors to create compounds with a variety of biological functions, such as anti-Alzheimer, antibacterial, antihemolytic, anti-inflammatory, anti-inflammatory, anti-tumor, and insecticide properties [171]. Some of the plant-derived secondary metabolites are mentioned in Table 5.

**Table 5:** Secondary metabolites production in PMF system.

Secondary Metabolites	Plants	Culture type	Reference
Acetylshikonin	*Echium plantagineum*	Hairy root	[170]
Alkaloids	*N. tabacum*	Transgenic	[169]
Diosgenin	*Trigonella foenum-graceum*	Hairy root	[168]
Artemisinin	*Aconitum heterophyllum*	Hairy root	[330]
baicalin, baicalein, wogonin	*Scutellaria baicalensis*	Hairy root	
Chlorogenic acid	*Platycodon grandiflorum*	Hairy root	
Diosgenin, Prolin	*Helicteres isora*	Hairy root	
Dopamine	*Portulaca oleracea*	Hairy root	
Emodin, Physcion	*Polygonum multiflorum*	Hairy root	
Glycyrrhizin	*Abrus precatoroius*	Hairy root	
Hydroxycinnamate	*Cichorium intybus*	Hairy root	
Mitragynine, Ursolic Acid	*Mitragyna speciosa*	Hairy root	
Podophyllotoxin	*Liunm mucronatum*	Hairy root	
Salidroside	*Rhodiola crenulata*	Hairy root	
Silymarin	*Silybum marianum*	Hairy root	

## 5 The rise of PMF products in today's market

Many r-proteins derived from plants have been produced, with a few of them bringing it to market. The shortened form of γ-zein, marketed under the brand name Zera® by Era Biotech in Spain, depends on fusing its N-terminal region with other proteins to trigger the production of protein bodies. These protein bodies based on γ-zein can be used to encapsulate r-proteins and exhibit immunostimulatory properties, which could be advantageous for the delivery of vaccines [172]. The higher expression level of recombinant β-Glucuronidases and egg white avidin derived from transgenic maize and commercially marked by Merck Merck via its subsidiary MilliporeSigma. The physiological characterization of maize-derived avidin exhibited an identical N-terminal sequence of egg white and antigenic similarities [173]. Taliglucerase alfa is the only biopharmaceutical product produced in plant cell suspension cultures that has been approved for commercialization thus far after the completion of phase I–III human trials and this recombinant human glucocerebrosidase currently marketed by the Israeli company Protalix Biotherapeutics [174]. Elelyso, a product of taliglucerase alfa, was introduced into the market in May 2012 after receiving FDA approval for injection use as an enzyme substitute for adult patients suffering from Gaucher's disease. Lectins, GF, and cytokines transiently produced in *N. benthamiana* for research reagents by iBio, Inc. COVIFENZ (SARS-CoV-2 virus-like particles) produced transiently in *N. benthamiana* for human vaccines by Medicago, Inc [175]. Pembrolizumab (“Keytruda” marketed by Merck, USA) and nivolumab (“Opdivo” marketed by Bristol-Myers Squibb, USA) are mAbs that are utilized in cancer immunotherapy to improve the immune response against cancer cells by targeting the T cell programmed cell death protein 1 (PD-1) receptor. These both have received approval for the treatment of different cancers and have demonstrated a notable improvement in survival rates as well as a reduced progression of the disease in patients with conditions like lung cancer, melanoma, and Hodgkin lymphoma [176]. These examples showcase the diverse range of PMF products that can be successfully developed using plant-based biopharmaceutical engineering systems.

## 6 Manufacturers in alternative food proteins development

PMF is an alternative method of producing protein that allows animal proteins to be produced in plants by combining precision fermentation techniques with plant agriculture. The list of publicly traded alternative protein firms is small but growing, with Moolec Science becoming the first plant molecular farming company to go public in early 2023. The newest product "Piggy Sooy" from Moolec produced a notably substantial amount of pork protein, marking an exceptional achievement in the company's Meat Replacements Program for the Soybean platform. Up to 26.6 % of the total soluble protein in soy seeds, the animal protein expressed itself at a high level, four times greater than the company had predicted. Although Moolec's soybeans are the same shade of pink as the pig, the outcome is readily apparent [177]. Recombinant lactoglobulin/casein in soybean is produced by Nobell Foods. They exhibit the fusion expression of lactoglobulin and casein, which can subsequently be cleaved upon isolation. These methods use plant expression systems to produce whey and casein, two important dairy protein constituents, at the same time [178]. Miruku also developing PMF systems for producing dairy proteins [179]. Growth factors for cultured meat medium and pharmaceuticals are produced by Tiamet Sciences and Bright Biotech currently working on the development of Transforming Growth Factor β3 and Fibroblast Growth Factor 2. PoLoPo is going to take the place of animal-based proteins in plants. With the help of their innovative SuperAA platform, each potato may become a mini bio-factory and produce animal-based proteins. Veloz Bio is a biotechnology company also developing animal proteins (e.g. egg proteins) in plants. The goal of BioBetter is to provide high-quality, cost effective recombinant bovine growth factors. BioBov FGF2, BioBov Insulin, and BioBov Transferrin are successfully produced and currently working to develop three more GF: BioBov TGFb1, BioBov IGF-1, and BioBov Activin A. An animal protein called chymosin, which is present in the intestines of goats and calves and may also be made by fermentation, is already produced by the Bioceres spinout firm using GM safflower [180]. Current manufactures and developers of PMF products are presented in Table 6.

**Table 6:** Commercial land scape of PMF products.

Company	Country	Products	Website
Vytrus Biotech	Spain	Cosmetic ingredient	https://www.vytrus.com/
Samabriva	France	r-proteins	https://samabriva.com/
Protalix BioTherapeutics	Israel	ELELYSO®, r-proteins	https://protalix.com/
PlantForm	Canada	Keytruda®, ranibizumab drug, PhD9 antibody, rBuChE, Hemopexin, Lucentis®	https://www.plantformcorp.com/
Plant Advanced Technologies	France	r-proteins, cosmetic	https://www.plantadvanced.com/
Planet Biotechnology	USA	Plantibodies (CMG2-Fc, DPP4-Fc)	https://www.planetbiotechnology.com/
Phyton Biotech	USA	Paclitaxel, Docetaxel	https://phytonbiotech.com/
ORF Genetics	Iceland	MESOkine, ISOkine, DERMOkine	https://www.orfgenetics.com/
Nomad Bioscience	Germany	Vaccines	https://www.nomadbioscience.com/
Medicago	Canada	Seasonal flu vaccine	https://www.medicagogroup.com/
Mapp Biopharmaceutical	USA	mAbs	https://mappbio.com/
Leaf expression systems	UK	Antibodies, VLPs, Enzymes	https://www.leafexpressionsystems.com/
KBio	California	mAbs, Vaccines	
Icon Genetics	Germany	Vaccines	https://www.icongenetics.com/
iBio Inc	USA	mAbs, Vaccines	https://ibioinc.com/
ZIP Solutions	Spain	Vaccines	https://zipsolutions.cat/
Diamante SRL	Italy	Vaccines	https://www.diamante.tech/
CollPlant	Israel	Collagen	https://collplant.com/
AntoXa	Canada	PhD9, rBuChE, Hemopexin	https://antoxacorp.com/
Agrenvec	Spain	Diagnostic antibodies and growth factors	https://www.agrenvec.es/

## 7 Bio-safety concerns of PMF products

**Fig. 2:**
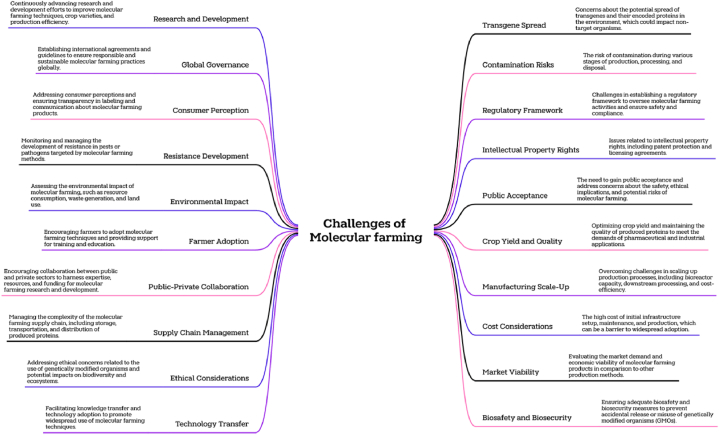
Challenges of PMF in plants include various aspects related to safety and production processes.

Several problems were noticed when thinking about edible vaccinations. It was unclear which classification the edible vaccines should fall under or which part of it-whether the antigen itself, genetically modified produce, or transgenic seeds-needs to be licensed. To make sure they don't get into the food supply, the regulatory organizations give it an extensive review [181]. There are specific problems with edible vaccines right now, and their future relies on several factors. The development of the edible vaccine is heavily reliant on public acceptance. The advantages and applications of edible vaccines should be known by society [182]. An edible vaccine offers a cost-effective, safe, and better disease prevention compared to conventionally developed vaccines. The use of MF techniques poses various biosafety issues, which include the spread of transgenes in the environment, the accumulation of r-proteins in the ecosystem, the contamination of food and feed chains with transgenes and related products, and the safety and quality of the products. Plant molecular farming brings up some new difficulties with factors like transgene amplification, diffusion, and accumulation of harmful r-proteins that might lead to the need for biosafety concerns about contamination of the environment [183]. Several methods are employed to minimize the potential risks associated with PMF. Physical containment is one such strategy, which entails growing GM plants inside of physical structures. Another strategy is called spatial confinement, which combines several approaches to minimize the spread of transgenes and their products. However, the large-scale manufacturing of biotechnology products sparks a debate on the topic of economic effect, waste management, biosafety, and bioethical concerns [184]. All molecular farming-related genetically modified plants, like all other GM plants, must undergo a thorough risk assessment before being allowed to be used in the field. The nature of the recombinant genes employed in PMF brings additional challenges and issues that might necessitate the addition of specialized biosafety considerations to the framework for evaluating the risk of GM plants used for food, feed, or processing. PMF unveils several challenges, including those pertaining to manufacturing procedures, biosafety, and product safety. Among the major obstacles are illustrated in Fig. 2.

PMF products to go through extensive evaluations of potential health and environmental risks prior to field testing, commercialization, and distribution. The evaluation makes sure they don't endanger the environment or public health. In Asia, the regulatory procedure for products derived from molecular farming which uses genetically modified organisms to create medications or other useful compounds can differ greatly from nation to nation. Regulatory agencies in each nation or area are usually in responsible for approving these kinds of products. US: The U.S. Food and Drug Administration (FDA) oversees and grants approval for plant-derived recombinant pharmaceuticals through its Centre for Biologics Evaluation and Research (CBER) and Centre for Drug Evaluation and Research (CDER). The US Department of Agriculture (USDA) controls the introduction and cultivation of GM plants into the environment through its Animal and Plant Health Inspection Service (APHIS) [185,186]; European Union: The European Medicines Agency (EMA) assesses and approves recombinant medicines derived from plants for use within the EU. The EMA's Committee for Medicinal Products for Human Use (CHMP) oversees the scientific evaluation process. The European Food Safety Authority (EFSA) assesses the safety of GMOs and goods made from them within the EU, including plant-derived medications [187,188]. India: GMOs and their products, including those used in MF, must be assessed, and approved by the Genetic Engineering Appraisal Committee (GEAC), which is housed under the Ministry of Environment, Forest and Climate Change and Department of Biotechnology (DBT), Review Committee on Genetic Manipulation (RCGM) under the Ministry of Science and Technology [189]. China: The Ministry of Agriculture and Rural Affairs (MARA) [190]; Japan: The Ministry of Agriculture, Forestry, and Fisheries (MAFF) and the Ministry of Health, Labour, and Welfare (MHLW), Ministry of Economy, Trade and Industry (METI), Ministry of Education, Culture, Sports, Science and Technology (MEXT), Ministry of Finance (MOF), Ministry of the Environment (MOE) [191]; South Korea: the Ministry of Food and Drug Safety (MFDS) and five central administrative agencies [192]; Taiwan: The Council of Agriculture (COA) is responsible for regulating GMOs and associated products [193]. Other Asian countries such Thailand, Malaysia, Indonesia, and others have their own regulatory bodies for assessing GMOs/GMO products [194,195].

## 8 Perspective and conclusion

Production of r-protein from plants has plenty of benefits, but industrial adoption is hampered by the fact that mammalian and other cell cultures have long existed and are very well understood in an industrial setting, which makes it difficult for plant-based productions to attain market interest. To tilt the scales in favor of sustainable PMF goods, it may be necessary to utilize the extra potential of PMF systems. Regarding the launch of protein products on a large-scale market, PMF is currently a niche platform. One of the biggest restrictions may be the huge cell size of plants and the consequently reduced number of protein synthesis factories per unit volume compared to mammalian and microbial systems. However, the PMF-produced proteins have huge distinct advantages, such as advanced glycan alteration mechanism, the ability to produce allergens and diagnostic proteins without the use of animal components or endotoxins (which is crucial for the creation of pharmaceuticals for some religious communities, vegans, and individuals with animal allergies), and the ability to produce therapeutics and mucosal vaccines at a reasonable cost because oral and topical administration does not require the use of high-cost downstream techniques. Future research must demonstrate that the above-mentioned advantages can be put into practice and scaled into an actual manufacturing process with associated by-products, which has not yet been done to create side streams that do not contain proteins. For further gains in process income, it will be critical to find innovative, mid-to-high-value by-products that can be produced economically from the leftover biomass. In contrast to traditional expression methods to produce non-pharmaceutical and pharmaceutical goods, plants have both techno-economic benefits. The many PMF systems, including chloroplast, nuclear and transient expression, and viral transfection systems, each have remarkable qualities that make it possible for them to develop a variety of diverse product “targets” while experiencing fewer production restrictions quickly and efficiently. The PMF platform has recently overcome several scientific and technological difficulties. Nevertheless, an important barrier to the plant system's broad adoption is the regulatory complexity connected to the manufacturing of therapeutic proteins. The non-pharmaceutical protein commercialization is simple and quicker owing to minimal regulatory problems because of the economical and higher scalability of PMF production techniques. Therefore, the global regulatory environment and constraints placed on items made from plants will have a notable impact on whether the technology is accepted by all people. A promising future for PMF biologics is suggested by the demand for r-proteins that are valuable for industry or pharmaceuticals, as well as by the system's established manufacturing capabilities and economic viability.

## 9 Search strategy

A systematic review was performed to explore the recombinant products produced in plant platforms. All pertinent research focusing on development strategies for PMF and discussing the PMF products such as mABs, vaccines, proteins, enzymes, pharmaceutical and nonpharmaceutical molecules and its developments. Select respected academic resources like PubMed, Scopus, Web of Science, Limaps, Consensus, and Google Scholar, as well as specialist databases related to the topic, such as Plant Cell Reports for plant molecular farming. Apply pertinent filters, such as publication date, language, and document type, to refine the results of your methodical searches in these databases by utilizing the defined keywords and Boolean operators. A range of keywords combinations were used in search terms, plant molecular farming, plant bioreactors, plant expression system, plantibodies, vaccine produced in plants, biopharmaceuticals, recombinant proteins and enzymes, transient expression system, PMF products. To find other studies, the reference lists of pertinent papers and reviews were also manually searched. Abstracts from conferences, patents, and organisational reports are examples of grey literature that should be included. The therapeutic roles of PMF products were also examined in observational studies, clinical trials, and experimental investigations. First, the relevance of the search results was screened, and then the eligibility of the complete text was evaluated. Selected studies' data were carefully extracted and thematically arranged.

## Funding

Not applicable.

## CRediT authorship contribution statement

**Jothi Kanmani Bharathi:** Writing – original draft, Methodology. **Preethika Suresh:** Methodology, Data curation. **Muthu Arjuna Samy Prakash:** Writing – review & editing, Validation, Supervision. **Sowbiya Muneer:** Writing – review & editing, Validation, Supervision, Funding acquisition.

## Declaration of competing interest

The authors declare no conflict of interest.
